# Benchmarking Guanidinium Organosulfonate Hydrogen-Bonded Frameworks for Structure Determination of Encapsulated Guests

**DOI:** 10.1021/acsmaterialslett.4c00400

**Published:** 2024-04-10

**Authors:** Anna Yusov, Alexandra M. Dillon, Mohammad T. Chaudhry, Justin A. Newman, Alfred Y. Lee, Michael D. Ward

**Affiliations:** † Department of Chemistry and Molecular Design Institute, New York University, New York City, New York 10003, United States; ‡ Analytical Research and Development, Merck & Co., Inc., 126 East Lincoln Avenue, Rahway, New Jersey 07065, United States

## Abstract

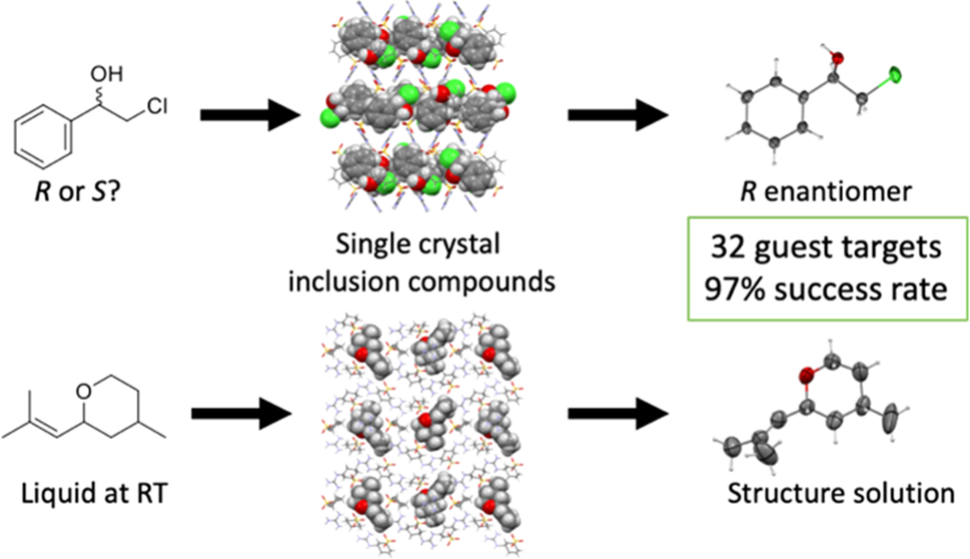

Single crystal X-ray diffraction (SCXRD) is arguably the most definitive method for molecular structure determination, but it is often challenged by compounds that are liquids or oils at room temperature or do not form crystals adequate for analysis. Our laboratory previously reported a simple, cost-effective, single-step crystallization method based on guanidinium organosulfonate (GS) hydrogen bonded frameworks for structure determination of a wide range of encapsulated guest molecules, including assignment of the absolute configuration of chiral centers. Herein, we expand on those results and report a head-to-head comparison of the GS method with adamantoid “molecular chaperones”, which have been reported to be useful hosts for structure determination. Inclusion compounds limited to only two GS hosts are characterized by low *R*_1_ values and Flack parameters, infrequent disorder of the host and guest, and manageable disorder when it does exist. The structures of some target molecules that were not included or resolved using the adamantoid chaperones were successfully included and resolved by the GS hosts, and vice versa. Of the 32 guests attempted by the GS method, 31 inclusion compounds afforded successful guest structure solutions, a 97% success rate. The GS hosts and adamantoid chaperones are complementary with respect to guest inclusion, arguing that both should be employed in the arsenal of methods for structure determination. Furthermore, the low cost of organosulfonate host components promises an accessible route to molecular structure determination for a wide range of users.

Single-crystal X-ray diffraction (SCXRD) has been instrumental for structure determination of inorganic compounds and organic molecules dating back to Bragg in 1913^1^ and Lonsdale and Whiddington in 1929.^2^ Structure determination was further advanced by Bijvoet et al. in 1951, with the first example of determination of absolute configuration by SCXRD.^3^ Although nuclear magnetic resonance spectroscopy can be used for structure elucidation, SCXRD is regarded as the most definitive method, including the assignment of the absolute configuration of stereogenic centers. Compounds that are liquids at room temperature or exist only as oils, however, generally do not form crystals suitable for conventional SCXRD. The “crystalline sponge method” (CSM) employs metal–organic frameworks as hosts for target molecules to circumvent these challenges. CSM, however, can be limited by fixed pore apertures that impose a limit on guest size, as well as low occupancy and disorder of guest and solvent molecules.^4−10^ Other methods for molecular structure determination include phosphorylated macrocycle hosts,^11^ the use of chiral MOFs,^12^ and alcohols converted to sulfates followed by crystallization with guanidinium ions.^13^ Recently, our laboratory reported the use of guanidinium organosulfonate (GS) hydrogen-bonded host frameworks for structure solution of encapsulated guests using a single-step solvent-based crystallization method, affording reliable assignment of absolute configuration and relative stereochemistry for guests with stereogenic centers,^14,15^ as well as providing a structural explanation for reactive pathways.^16^ Later, substituted tetraaryladamantanes, a.k.a. “molecular chaperones”,^17−22^ were employed as hosts for the determination of molecular structure, including absolute configuration, for a variety of guest molecules. This method involved adding one of three different adamantoid chaperones (Figure S33) to a neat guest, in liquid form, at room temperature, heating the mixture to achieve a homogeneous solution, followed by cooling with the aim of producing a cocrystal of the adamantoid chaperone and guest.^17,19,21^ Subsequently, other research groups reported the use of GS frameworks for structure determination of target molecules,^23,24^ and very recently, a report in this journal described an anthracene-modified adamantoid molecular chaperone for structure determination of guests.^25^

Over the past three decades, our laboratory has reported a substantial number of molecular host frameworks, built from two-dimensional hydrogen-bonded networks of guanidinium (G) and organosulfonate (S) ions (Figure 1), capable of encapsulating a wide range of guests and determining their molecular structure.^26−30^ These frameworks can adopt various architectures and pucker about hydrogen-bonding “hinges” in response to the steric demands of a target guest, enabling inclusion of a wide range of guests having various sizes and shapes in stoichiometric amounts. Framework puckering, combined with free motion of the organosulfonate residue, allows a GS host to “shrink-wrap” about the guest molecules, facilitating dense packing with associated mitigation of guest disorder. Target molecules can be included in the frameworks through a simple single-step crystallization in which a small amount of the target molecule is added to a solution of the GS framework components at room temperature. The chemical and structural diversity of organosulfonates allows for tailoring of the size, shape, and environment of the inclusion cavities.^29,31^ Approximately 500 GS inclusion compounds with well-characterized single crystal structures have been reported with more than 150 unique guests.^28,29,32^ Furthermore, the presence of sulfur atoms provides stronger anomalous scattering, which is advantageous for the assignment of an absolute configuration for target molecules with only lighter elements.

**Figure 1:**
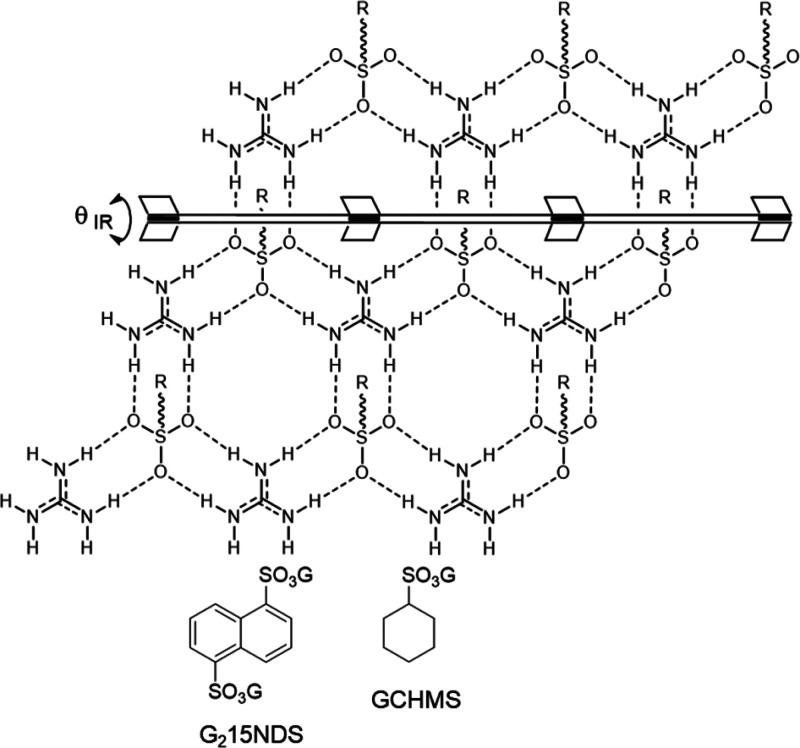
Top: The typical quasihexagonal GS sheet (top), illustrating the hydrogen-bonded “hinge” that allows facile puckering of the GS sheet allowing for “shrink-wrapping” around guests in the inclusion cavities. The R substituents on the sulfur atoms denote organic groups that can project from either side of the GS sheet. Bottom: The two guanidinium organosulfonate hosts used here.

Recognizing that the attributes of the adamantoid chaperones and GS hosts may prove to be complementary with respect to structure determination, we attempted the crystallization of GS host frameworks with 32 guest targets (Figure 2). Of these 32 targets, 23 were selected from 53 attempted for the three originally reported adamantoid chaperones^17,21^ to allow for a head-to-head comparison of the two kinds of hosts. We chose to measure the performance of GS hosts against the adamantoid chaperones, because the latter employed a comprehensive library for guest inclusion and structure refinement, providing a rare opportunity for a comprehensive head-to-head comparison. Although the guest molecules were generally of low molecular weight, such compounds are often synthetic targets or intermediates that require a structure determination or confirmation. Our investigation was limited to 23 targets from the group for reasons of cost and availability. The remaining nine targets of the 32 are reported here to illustrate further the utility of the GS hosts. Collectively, the 32 targets span polar to nonpolar and aliphatic to aromatic, contain various functional groups, and have wide-ranging shapes and sizes. Although our laboratory has reported inclusion in GS hosts based on more than 100 organosulfonates,^33^ we limited our investigation to only two GS hosts—guanidinium 1,5-naphthalene disulfonate (G_2_1,5-NDS) and guanidinium cyclohexane monosulfonate (GCHMS)—to ensure a fair comparison with the three adamantoid chaperones.^17^ The occupancy of guest molecules in the GS inclusion compounds was stoichiometric, and disorder, if it exists, was manageable generally. The findings described herein show that the adamantoid and GS hosts are complementary with respect to the inclusion of target guest molecules and determination of their structure, which includes definitive assignment of the absolute configuration for guests with stereogenic centers.

**Figure 2:**
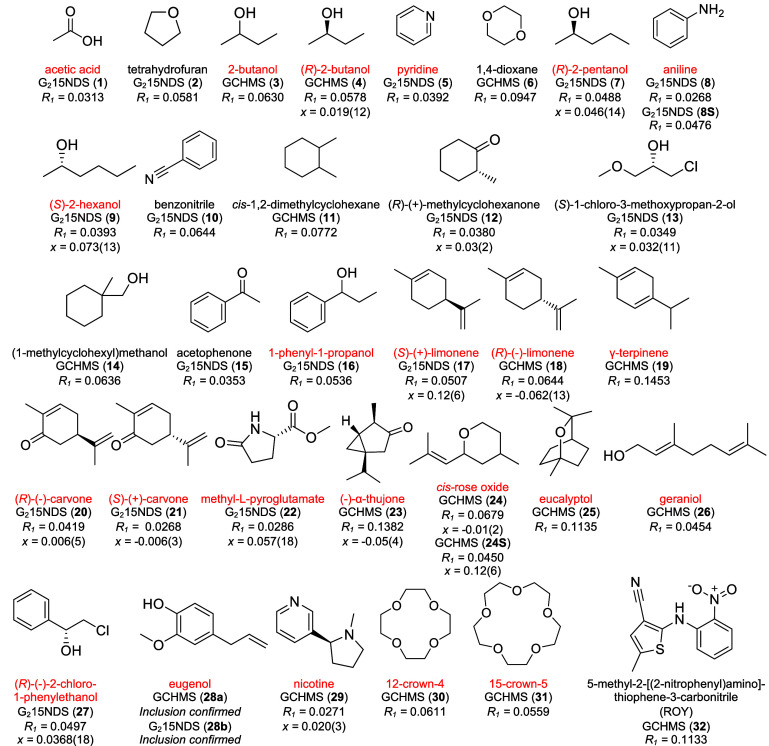
Target guest molecules included and fully refined in GCHMS or G_2_1,5-NDS hosts, listed in order of increasing molecular weight. The GS framework corresponding to each target molecule and the *R*_1_ values are provided. Flack parameters (*x*) are provided for structures with chiral guests. The near-zero value and high precision of the Flack parameters for inclusion compounds provide confidence in the assignment of the absolute configuration. Guests denoted in red correspond to those investigated with “molecular chaperones”.^17,21^ Compound **5** contains both stereoisomers of *cis*-rose oxide in equal amounts; the stereochemistry is not denoted here for the sake of clarity. The structure of eugenol in **28a** and **28b** could not be resolved due to guest disorder. Examples **8S** and **24S** denote data collected using synchrotron radiation. Examples of the structure determination of 21 other guests (not shown here) using GS hosts have been reported elsewhere.^14−16,23,24^

Inclusion compounds with the G_2_1,5-NDS host adopt a 1:1 host:guest stoichiometry (Table S5), even though the guest volumes, calculated using a simple and accurate formula,^34^ are wide-ranging, from 58 Å^3^ (acetic acid, **1**) to 170 Å^3^ ((*R*)-(−)-carvone, **20**; (*S*)-(+)-carvone, **21**). This can be attributed to the structural compliance of the GS host, which enables the host framework to “shrink-wrap” around the target guest molecules. The G_2_1,5-NDS inclusion compounds adopt the usual and previously observed simple brick architecture, which is illustrated in Figure 3 for G_2_1,5-NDS⊃(*R*)-2-pentanol (**7**), with the exceptions of those with acetic acid, tetrahydrofuran, pyridine, aniline, benzonitrile, and methyl-*L*-pyroglutamate guests. G_2_1,5-NDS⊃acetic acid (**1**), G_2_1,5-NDS⊃pyridine (**5**), and G_2_1,5-NDS⊃methyl-*L*-pyroglutamate (**22**) adopt a puckered simple brick architecture in which the GS sheets are sustained despite hydrogen bonds with the target molecules (Figure S34). In the case of compound **1**, the acetic acid guests exist as hydrogen bonded dimers nestled inside the framework cavities. G_2_1,5-NDS⊃aniline (**8**) exhibits a bilayer-type architecture with hydrogen bonding between the guest and the GS sheet, with a water molecule bridging adjacent GS sheets (Figure S35), which has been previously observed.^16^ G_2_1,5-NDS⊃benzonitrile (**10**) exhibits a bilayer architecture, but hydrogen bonds between the nitrile group of the guest and the G_2_1,5-NDS host are absent. G_2_1,5-NDS⊃tetrahydrofuran (**2**) adopts a unique layered architecture where hydrogen bonds between the guanidinium and tetrahydrofuran molecules results in a one-dimensional ribbon in lieu of the usual GS sheet (Figure S36). Inclusion compounds **1**, **2**, **5**, **8**, **10**, and **22** illustrate the tolerance of the GS frameworks and the hydrogen bonded sheet to guest molecules with hydrogen-bond donor and acceptor character.

**Figure 3:**
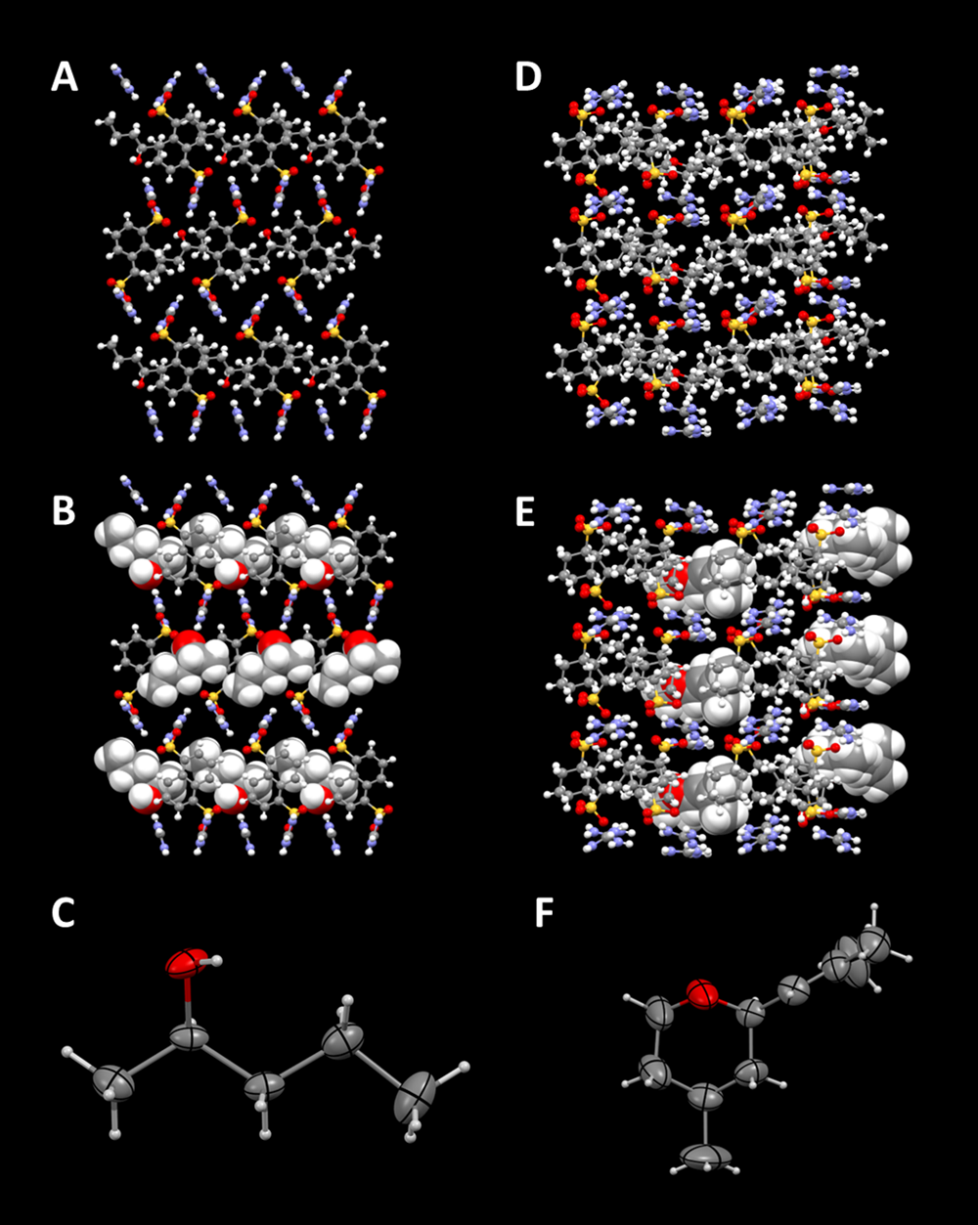
Illustrative crystal structures. (A–C) (G_2_1,5-NDS)⊃(*R*)-2-pentanol (**7**) and (D–F) (GCHMS)_4_⊃*cis-*rose oxide (**24**), depicted as ball-and-stick (top), target guest molecules as space filling (middle), and guests with ellipsoids at 50% probability (bottom).

The GCHMS host readily formed inclusion compounds with a variety of layered architectures, some new and unanticipated, in which the GS sheet is preserved except for 15-crown-5 (**31**), which is incorporated into the hydrogen bonded sheet through hydrogen bonding between its oxygen atoms and guanidinium protons. The pattern of the projections of the organic residues above and below each GS sheet is guest-dependent, however. This is reminiscent of previously reported inclusion compounds formed from a large collection of guanidinium arenemonosulfonates and aromatic guests that produced more than 300 inclusion compounds and four different architectures, each described by GS sheets with a unique “up–down” projection topology.^27^ The facility of guanidinium monosulfonates for encapsulating guests can be attributed to the absence of a constraint on the registry of adjacent sheets, which provides a degree of freedom for molecular packing between the GS sheets that is not available to disulfonates. GCHMS, reported here for the first time, is particularly adept with respect to trapping aliphatic target molecules, as illustrated for (GCHMS)_4_⊃*cis-*rose oxide (**24**) in Figure 3. Details of the GCHMS architectures will be reported elsewhere.

Of the 23 guests in the head-to-head comparison, 17 were included and refined in the adamantoid chaperones, whereas 22 were included and refined in the GS hosts. The lone exception was eugenol, which was included but could not be refined (compounds **28a** and **28b**). In most cases, the inclusion of a target guest in both GS hosts was not attempted if successful guest refinement was achieved in one of them (Table S3). The GS inclusion compounds generally afforded better *R*_1_ values, improved Flack parameters where appropriate, and smaller thermal ellipsoids (Tables S3, S4). Inclusion of acetic acid, γ-terpinene, methyl-*L*-pyroglutamate, *cis-*rose oxide, eucalyptol, and 15-crown-5 in the adamantoid hosts was either not successful, or inclusion was confirmed but satisfactory refinement was not obtained.^17^ These guests, however, were included by either G_2_1,5-NDS or GCHMS hosts (compounds **1**, **19**, **22**, **24, 24S**, **25**, and **31**) and satisfactory refinements were obtained for the corresponding inclusion compounds. Data for two inclusion compounds with aniline (**8**, **8S**) and *cis-*rose oxide (**24**, **24S**) guests were collected by using synchrotron radiation as well as on a conventional SCXRD instrument (Table S3). The synchrotron and laboratory data sets both provided low *R*_1_ values and good data quality, demonstrating that access to synchrotron data can improve data quality but the use of a conventional SCXRD instrument is sufficient.

GS hosts have a high density of sulfur atoms, which improves anomalous scattering required for reliable absolute configuration determination. This is illustrated by G_2_1,5-NDS⊃(*R*)-2-pentanol (**7**; Figure 3), for which the Flack parameter was *x* = 0.046(14). In comparison, the Flack parameter for the adamantoid inclusion compound TEO⊃(*R*)-2-pentanol (TEO = tetrakis(2,4-diethoxy-phenyl)adamantane; Figure S33), in which oxygen as the strongest scatterer, was *x* = −0.6(6) (CSD refcode: ZURXIW).^17^ One of the three adamantoid chaperones (Figure S33) contains a heavy atom (bromine), but no inclusion compound for (*R*)-2-pentanol was reported for this host, precluding a direct comparison. Of the six reported inclusion compounds using this adamantoid chaperone, only one contained a chiral guest, muscone (CSD refcode: ZURXAO), but the Flack parameter was *x* = 0.523(19) and absolute configuration could not be assigned.^17^

The guests included in the GS frameworks described herein are liquids or oils at room temperature, with the exception of ROY (named for its Red, Orange, and Yellow crystal polymorphs^35−39^). GCHMS⊃ROY (**32**) crystallized as red-orange plates, enabling convenient discrimination of the inclusion compound from guest-free GCHMS crystals. The ROY guest is planar (Figures S37, S38), unlike other GS inclusion compounds containing ROY.^40^ Moreover, the planar conformation does not exist in any of the 13 polymorphs of ROY for which single crystal structures have been determined, although near-planarity was observed in the “R18” polymorph.^35−39,41^ This example further demonstrates the ability of the GS frameworks to encapsulate solid guest targets, like many others reported previously.^14,28,42−46^

The complementary nature of the GCHMS and G_2_1,5-NDS hosts can be illustrated with three guest targets (limonene, geraniol, and eugenol). Inclusion of (*S*)-(−)-limonene and (*R*)-(+)-limonene was confirmed in both G_2_1,5-NDS and GCHMS, and satisfactory refinement was achieved for (*S*)-(−)-limonene and (*R*)-(+)-limonene in G_2_1,5-NDS (**17**) and GCHMS (**18**), respectively. Inclusion of geraniol was successful with G_2_1,5-NDS, but the guest structure could not be resolved due to substantial positional disorder along a solvent channel. Inclusion and full refinement were achieved, however, by using the GCHMS host (**26**).

Eugenol was included stoichiometrically in both the GCHMS (**28a**) and G_2_1,5-NDS (**28b**) hosts, but its structure could not be resolved due to guest disorder. The stoichiometry (2:1 host:guest in **28a** and 1:1 in **28b**) was assigned based on the electron density of the included guest. This type of disorder was not entirely unexpected, as it has been observed previously for linear triglyme guests in the channels of the G_2_1,5-NDS host (CSD refcode: TUQPIE).^47^ These examples illustrate that inclusion and structure refinement of almost all target guests were possible using only the GCHMS and G_2_1,5-NDS hosts. Satisfactory structure determination of 31 of the 32 guests in Figure 2 was achieved with at least one of these two hosts.

The adamantoid and GS hosts each have unique benefits and challenges, making them complementary with respect to inclusion and structure determination. The GS inclusion compounds are grown readily by adding a guest to a solution (typically methanol and/or ethanol) of the GS host, affording versatility with respect to including solid guests as well as liquids. The typical adamantoid host protocol requires a guest to act as the solvent to dissolve the adamantoid chaperones prior to crystallization, precluding the encapsulation of solid guests. Inclusion of a solid guest was achieved by evaporation of solvent containing the chaperone and guest.^20^ Moreover, heating is required to dissolve the adamantoid chaperone (up to 150 °C), which can be problematic for thermally sensitive targets. The crystallization of the GS inclusion compounds is achieved at room temperature via slow evaporation, which may be essential for thermally sensitive targets. The GS method requires several days for inclusion compound crystallization, whereas the adamantoid chaperones were reported to form inclusion compounds in 16 hours or less.^17^ Guests that are sensitive to acidic environments or the crystallization solvent may decompose or transform during the GS protocol. For example, attempts to grow GS inclusion compounds containing (*S*)-epichlorohydrin instead produced crystals of G_2_1,5-NDS⊃(*S*)-1-chloro-3-methoxypropan-2-ol (**13**) because of reaction with methanol solvent (Scheme S1). In contrast, unreacted (*S*)-epichlorohydrin was included by an adamantoid host, and its structure was determined (CSD refcode: ZURROW).^17^ GS hosts can form solvates and guest-free phases, which can compete with their crystallization with molecular targets. The adamantoid chaperones also form guest-free crystalline phases,^17,22,48^ which were observed during attempts to grow inclusion compounds of certain host–guest combinations. Like many GS inclusion compounds, solvate- and guest-free phases of G_2_1,5-NDS and GCHMS often have crystal habits distinct from their inclusion compounds, enabling identification of guest-containing crystals. The adamantoid chaperone protocol requires a substantial amount of the guest target molecule (up to 60 mg)^17^ to dissolve the chaperone, whereas inclusion in GS hosts can be realized with as little as 1 mg of the target guest,^16^ which is critical when guest quantities are limited. The synthesis of the chaperone molecules is more challenging than the synthesis of organosulfonates. Like other GS hosts, G_2_1,5-NDS and GCHMS can be prepared from a simple, single-step reaction from affordable and commercially available starting materials. Moreover, the chaperones are expensive (*ca*. US $1000 for 100 mg, as offered by Bruker AXS^49^), nearly 50 times the cost of the most expensive organosulfonate used (*ca*. $20 for 100 mg^50^), and the more recently reported anthracene-modified adamantoid chaperone is not commercially available. The combination of synthetic ease, commercial availability, and affordability promises accessibility of the GS hosts to a wide range of users.

In conclusion, two GS hosts, G_2_1,5-NDS and GCHMS, were used to encapsulate 32 guest molecules, all but one as a liquid at room temperature, to form crystalline inclusion compounds. X-ray diffraction permits satisfactory refinement of the inclusion compounds and their respective molecular guests with low *R* factors, limited disorder, and Flack parameters suitable for the definitive assignment of stereochemistry for guests with stereogenic centers. The GS sheet is tolerant of guests with hydrogen bond donors and acceptors, and in several examples the guest is anchored to the GS sheet through hydrogen bonding, an additional mechanism for reducing disorder. The GS system exhibited a high success rate 31 of 32 guests attempted, 97%, for inclusion and successful structure refinement of the guest molecules despite using only two GS hosts. Of the 23 guests in a head-to-head comparison, 17 were included and refined in the adamantoid molecular chaperones, whereas 22 were included and refined in the GS hosts, arguing for the use of both host systems in the arsenal of crystallization methods for structure determination of guest molecules using single crystal X-ray diffraction. Notably, except for eugenol, the two GS hosts outperform the anthracene-modified adamantoid chaperone^25^ with respect to inclusion and structure refinement for the seven guests common to both. Furthermore, the GS method is characterized by low cost, commercial availability, and facile synthesis of the organosulfonate host components, an absence of host disorder in most inclusion compounds, and manageable guest disorder when it exists. Moreover, more than 100 organosulfonates are readily available for use as hosts, promising to expand the utility of GS hosts even further.

## Supporting Information Available

Crystallographic data, X-ray characterization details including the constraint and restraints, experimental procedures, X-ray tables, comparisons to previously published data, crystal structures of inclusion compounds from X-ray data, thermal ellipsoids for refined guests, and crystal images (PDF)
Crystallographic information files (CIF)

## Supplementary Material

tz4c00400_si_001.pdf

tz4c00400_si_002.cif

## Accession Codes

Crystallographic data of these structures, including cif, res, fcf, and hkl files, have been deposited with the Cambridge Crystallographic Data Centre with Numbers 2307188–2307219, 2308623, 2310649, 2312929. Copies of these data can be requested, free of charge, from the CCDC Web site at https://www.ccdc.cam.ac.uk/structures/
