# Supplementary material for: Benchmarking Guanidinium Organosulfonate Hydrogen-Bonded Frameworks for Structure Determination of Encapsulated Guests
Source: ACS Mater Lett. 2024 Apr 10;6(5):1906–12. doi: 10.1021/acsmaterialslett.4c00400 (PMC11077584; doi:10.1021/acsmaterialslett.4c00400)
Supplement: Supplementary file 1 — tz4c00400_si_001.pdf [file tz4c00400_si_001.pdf]

Benchmarking guanidinium organosulfonate hydrogen-bonded frameworks for structure determination of encapsulated guests

Anna Yusov,[1] Alexandra M. Dillon,[1] Mohammad T. Chaudhry,[2] Justin A. Newman,[2] Alfred Y. Lee,[2,*] and Michael D. Ward[1,*]

[1]*Department of Chemistry and Molecular Design Institute, New York University, New York City, New York 10003, United States*

[2]*Analytical Research and Development, Merck & Co., Inc., 126 East Lincoln Avenue, Rahway, New Jersey 07065, United States*

**email: alfred.lee@merck.com, mdw3@nyu.edu*

Supplementary information

Contents

Materials and Methods

Table S1. Summary of constraints/restraints used in structure refinement.

Table S2. Detailed crystallographic data.

Figure S1-S32. Molecular structure of **1-32** depicted as ellipsoids with 50% probability.

Figure S33. Molecular illustration of the adamantoid chaperones.

Table S3. Head-to-head comparison of inclusion behavior, R factors and Flack parameters for crystalline inclusion compounds derived from GS hydrogen-bonded hosts and adamantoid molecular chaperones and guest molecules common to both.

Table S4. Comparison of atomic ellipsoids for GS host included guests against adamantoid based hosts.

Table S5. Inclusion compounds with $G_2$1,5-NDS, guest volumes, architectures, and puckering angles.

Table S6. Inclusion compounds with GCHMS, guest volumes and host:guest stoichiometries.

Figure S34. Crystal structures of disrupted simple brick architecture examples **1**, **5**, and **22**.

Figure S35. Crystal structure of disrupted bilayer architecture example **8**.

Figure S36. Crystal structure of unique architecture example **2**.

Figure S37. Molecular illustration of 5-methyl-2-[(2-nitrophenyl)amino]thiophene-3-carbonitrile (ROY).

Figure S38. Crystal structure of inclusion compound **32**.

Scheme S1. Scheme for the reaction of (*S*)-epichlorohydrin with methanol, affording (*S*)-1-chloro-3-methoxypropan-2-ol.

References

Materials

Cyclohexane sulfonic acid and 1,5-naphthalenedisulfonic acid tetrahydrate were purchased from Sigma-Aldrich (St. Louis, MO) at a (*ca.* price of \$167 USD per 1 gram,[1] and \$66.10 USD per 100 grams,[2] respectively).

Guests targets: acetic acid, tetrahydrofuran, 2-butanol, pyridine, dioxane, *cis*-1,2-dimethylcyclohexane, (R)-(+)-methylcyclohexanone, acetophenone, 1-phenyl-1-propanol, (*S*)-(-)-limonene, (*R*)-(+)-limonene, (*R*)-(-)-carvone, (*S*)-(+)-carvone, (-)-α-thujone, and 15-crown-5 were purchased from Sigma Aldrich. Guest targets: (*R*)-2-pentanol, (*S*)-2-hexanol, (*S*)-epichlorohydrin, geraniol, eugenol, and nicotine were purchased from VWR (Randor, PA). Aniline and methyl-L-pyroglutamate were purchased from Fisher (Hampton, NH). (*R*)-2-butanol and 12-crown-4 were purchased from Aaron Chemicals (San Diego, CA). *cis*-rose oxide and eucalyptol were purchased from 1PlusChem. Gamma-terpinene was purchased from A2B Chem (San Diego, CA). I-(-)-2-chloro-1-phenylethanol was purchased from BLD Pharmatech (Hyderabad, India). 5-Methyl-2-[(2-nitrophenyl)amino]-3-thiophenecarbonitrile was purchased from Ambeed (Arlington Heights, IL). All reagents were used as obtained with no additional purification.

Guanidinium organosulfonate apohosts were prepared by combining acetone solutions of approximately 1.10 molar equivalents of guanidinium tetrafluoroborate and 1.0 molar equivalents of a select organosulfonic acid to produce a precipitate of the corresponding guest-free apohost of the GS compound. The mixture was dried in a rotary evaporator and the resulting solid washed with acetone several times and dried in vacuo, affording the apohost of the GS compound.

Methods

Characterization methods. Crystals were mounted on a MiTeGen MicroMount with Type B immersion oil (Cargille Labs). Single crystal X-ray diffraction data was obtained using several instruments. Single-crystal X-ray diffraction data for **3**, **4-9**, **12-15**, **22** and **30** were collected at 100 K on a Bruker SMART APEX II diffractometer equipped with a PHOTON-II-C14 detector. The X-ray beam generated from an INCOTEC micro-focused Mo source was monochromated and collimated by a Montel multilayer optics. The wavelength from the Mo Kα radiation is 0.71073 Å. Crystal temperature was controlled by an Oxford Cryosystems 700+ Cooler. For each crystal, a phi scan (APEX4) was performed to evaluate the crystal quality and determine the data collection parameters. Full datasets were collected with omega scan methods. The data sets were processed with the INTEGRATE program of the APEX4 software for reduction and cell refinement.[3] Multi-scan absorption corrections were applied by the SCALE program for the area detector. Single-crystal X-ray diffraction data for **1**, **2**, **4**, **10**, **11**, **16**, **17, 28a**, **28b** and **31** were collected at 100 K using a Rigaku XtaLAB synergy instrument equipped with graphite monochromated copper Kα (λ = 1.5406 Å) radiation and a HyPix-6000HE detector. Unless otherwise specified, data were collected at a resolution of 0.837 Å, as per IUCr guidelines. Data was processed using CrysAlisPro V 1.171.42.54A, and absorption corrections were done using spherical harmonics or through face indexing using SCALE3 ABSPACK. Single crystal X-ray diffraction data for **29** were collected at 100 K on a Rigaku XtaLAB Synergy R (DW system) equipped with a HyPix-Arc 100 Hybrid Pixel Array Detector. The X-ray beam was generated by VariMax DW dual-wavelength confocal optics. Sample temperature maintained at 100 K with the Oxford Cobra open-flow cooler, and samples were mounted on nylon loops from Hampton Research. Single crystal X-ray diffraction data for **19-21**, **23-27** and **32** were collected at 130 K on a Bruker D8 VENTURE diffractometer using Cu Kα radiation. Crystal temperature was controlled by an Oxford Cryosystems 700+ Cooler. Single crystal X-ray diffraction data for **8S** and **24S** were collected at 100 K at the ChemMatCARS beamline of the Advanced Photon Source at Argonne National Laboratory using a Bruker APEX II CCD detector and an Oxford Cryojet Cooler. A double monochromatic incident X-ray beam of 0.2 mm × 0.2 mm in size was used, conditioned using Si(111) and Si(311) monochromators to have an energy of 30 keV (λ = 0.41328 Å). The detector was mounted orthogonally to the beam path with a sample-to-detector distance of 80 mm. The structures were solved by intrinsic phasing methods (SHEL-XT or SHEL-XS) and the structure models were completed and refined using the full-matrix least-square methods on F2 (SHELXL).[4,5] Non-hydrogen atoms in the structures were refined with anisotropic displacement

parameters, and hydrogen atoms on carbons were placed in idealized positions using riding models. OLEX2 solvent mask was used to omit heavily disordered guest molecules that could not be refined in compounds **28a** and **28b**.[6] Graphical representation of crystallographic data was done using Mercury (Version 4.3.1).[7] Crystallographic data of these structures, including cif, res, fcf, and hkl files, have been deposited with the Cambridge Crystallographic Data Centre with Numbers **2307188-2307219**, **2308623**, **2310649**, **2312929**. Copies of these data can be requested, free of charge, from the CCDC website at https://www.ccdc.cam.ac.uk/structures/.

Crystallographic constraints and restraints. Restraints and constraints were employed during structure refinement of some inclusion compounds. The restraints/constraints used for each of these compounds are provided below in Table S1. The rationales for their use are as follows:

RIGU: This global bond restraint was applied to select structures in order to cause the bonds to behave more rigidly. An earlier version of this command, DELU, was used in certain cases based on crystallographer preference.

SIMU: The thermal similarity restraint, SIMU, was employed for atoms within a motif, particularly in disordered groups, to achieve comparable thermal parameters and to control variance based on standard deviation. This restraint was mainly applied to minor disordered groups where connected atoms displayed unrealistic thermal displacement.

ISOR: This command, which causes thermal ellipsoids (atomic displacement factors) to behave more spherically, was only used in cases wherein disordered atoms were overlapping with others. ISOR was used cautiously due to its restrictive nature.

EADP: This thermal similarity command causes two or more ellipsoids to have identical thermal parameters. EADP can be an aggressive tool and it was used sparingly, primarily when SIMU failed to yield chemically reasonable results (rigid bond requirements), often for disordered atoms overlapping on symmetry elements.

SADI: SADI is a bond length similarity restraint that causes pairs of atoms to have comparable distances (the difference is controlled by the standard deviation). SADI was applied frequently to maintain chemical consistency in disordered groups and to maintain bond lengths within motifs.

DFIX: Similar to SADI, except the bond length is fixed to a certain value (varied by standard deviation). DFIX was used in cases where disorder was present and bond lengths could not be controlled reliably with SADI.

DANG: This 1,3-bond distance restraint was used to limit the distance between non-bonding groups (variance controlled by standard deviation). DANG was used in disordered structures to render the geometry of motifs chemically reasonable.

EXYZ: This command used to fix the coordinates of two or more atoms to be identical in disordered structures wherein the atoms occupy the same location.

FLAT: This command was used for phenyl rings that were disordered and did not behave as planar molecules.

Solvent Mask: In the few cases in which guest disorder precluded accurate modeling, the Olex2 solvent mask was used to remove electron density from the refinement model.

TWIN: This command adds a twin matrix to twinned structures, often in conjunction with a batch scaling factor, BASF.

Table S1. Summary of constraints/restraints used in structure refinement.

Structure	RIGU	SIMU	ISOR	EADP	SADI	DFIX	DANG	EXYZ	FLAT	Solvent Mask	TWIN
1											
2											
3			x	x		x	x				
4	x				x		x				
5											
6											x
7						x	x				
8											
8S						x	x				
9				x		x	x	x	x		
10											
11	x		x			x					
12											
13			x			x					
14	x		x			x	x				
15											
16				x		x	x		x		
17	x			x							x
18	x	x	x								
19	x	x	x	x	x						
20											
21											
22						x					
23	x	x		x	x	x		x			
24	x			x	x						
24S				x		x					
25	x	x			x						
26	x			x							
27	x		x	x	x						
28										x	
29	x		x								
30				x							x
31											
32	x	x				x					

Crystallization methods. Crystals of **1** were grown via slow evaporation from a solution comprising methanol (1 mL), acetic acid (100 µL) and 10 mM guanidinium organosulfonate (GS) apohost. Crystals of **10** were grown via slow evaporation from a solution comprising methanol (1 mL), benzonitrile (100 µL) and 10 mM guanidinium organosulfonate (GS) apohost. Crystals of **2** were grown by vapor diffusion of THF into a solution of apohost (10 mM) in methanol (1 mL). Crystals of **17** were grown in a 1:1 MeOH:isopropyl alcohol solution (1mL) with apohost (10 mM) and target guest (10 mg). Single crystals of inclusion compounds **3-9** and **11-31** were obtained by evaporation of a 2:1 solution of methanol:ethanol (1 mL) containing the apohost (5 mM) and the target guest molecule (10 mg). Crystallizations were performed in a 20 mL scintillation vial under ambient temperature over a period of one to five days.

Single crystals of **32** were obtained by slow evaporation of a 1:1 solution of methanol:acetonitrile containing 5-Methyl-2-[(2-nitrophenyl)amino]-3-thiophenecarbonitrile (ROY) (1.3 mg in 500 µL of acetonitrile) and the GCHMS apohost (1.1 mg in 500 µL of methanol). Crystallization was performed in a 20 mL scintillation vial, and the inclusion compound was grown via slow evaporation over several days.

Volume calculations. Molecular volumes were calculated by the formula published by Zhao et al.[8]

Table S2. Detailed crystallographic data.

**Compound name**	$G_2$1,5-NDS⊃acetic acid	$G_2$1,5-NDS⊃ tetrahydrofuran	GCHMS⊃2-butanol	GCHMS⊃(*R*)-2-butanol
**Structure file name**	**1**	**2**	**3**	**4**
**X-ray lab code**	mds104	mds022	23mdw57ay	mds192
**CCDC no.**	2307191	2307188	2307206	2307219
**Formula by X-ray**	$C_{32}H_{52}N_{12}O_{20}S_4$	$C_{16}H_{26}N_6O_7S_2$	$C_{18}H_{44}N_6O_7$S2	$C_{18}H_{44}N_6O_7S_2$
**Formula weight**	1053.09	478.55	520.71	520.71
**Crystal habit**	Clear colorless rhombohedral	Clear colorless block	Clear colorless plate	Clear colorless irregular
**Crystal size (mm)**	0.38 x 0.34 x 0.25	0.31 x 0.04 x 0.03	0.480 x 0.410 x 0.060	0.24 x 0.15 x 0.05
**Crystal system**	Orthorhombic	Triclinic	Monoclinic	Monoclinic
**Space group (no.)**	*Pbam* (55)	*P-1* (2)	$P2_1/n$ (14)	$P2_1$ (4)
a **(Å)**	19.5544(4)	7.5182(3)	7.1238(5)	7.1236(2)
b **(Å)**	7.8928(2)	11.5511(4)	14.8642(10)	14.8587(4)
c **(Å)**	7.32820(10)	13.0958(4)	12.7990(9)	12.8150(3)
α **(°)**	90	101.241(3)	90	90
β **(°)**	90	98.238(3)	93.933(4)	94.133(2)
γ **(°)**	90	99.034(3)	90	90
V **(Å^3)**	1131.03(4)	1083.74(6)	1352.09(16)	1352.91
Z	1	2	2	2
D_c **(g cm^{-3})**	1.546	1.466	1.279	1.278
***F*(000)**	552	504	564	564
μ **(mm^{-1})**	2.736	2.687	0.243	2.181
**Total reflections**	5798	19392	9903	24367
**Unique reflections**	1187	4292	5434	15482
R_{int}[d]	0.0304	0.0437	0.0558	n/a
R_1[a] **[*I* > 2 ʃ(*I*)]**	0.0313	0.0581	0.0630	0.0578
wR_2[b] **(all data)**	0.0831	0.1669	0.1392	0.1683
**GOF (all data)**	1.074	1.062	1.125	1.095
**Flack parameter**[c]	n/a	n/a	n/a	0.019(12)
**Restraints**	0	0	42	204
**Parameters**	99	280	192	313

[a]$R_1 = \Sigma||F_o|-|F_c||/\Sigma|F_o|$; [b]$wR_2=\{\Sigma[w(F_o^2-F_c^2)^2]/\Sigma[w(F_o^2)^2]\}^{1/2}$; [c]n.a. – not applicable; [d]n/a – not applicable because the structure is twinned.

Table S2 (continued). Detailed crystallographic data.

Compound name	G_21,5-NDS ⊃pyridine	GCHMS⊃dioxane	G_21,IDS⊃(*R*)-2-pentanol	G_21,5-NDS⊃aniline
**Structure file name**	**5**	**6**	**7**	**8**
**X-ray lab code**	23mdw30ay	23mdw22d	23mdw23ay	23mdw66ay
**CCDC no.**	2307201	2307211	2307204	2307202
**Formula by X-ray**	$C_{22}H_{28}N_8O_6S_2$	$C_{18}H_{42}N_6O_8S_2$	$C_{17}H_{30}N_6O_7S_2$	$C_{18}H_{27}N_7O_7S_2$
**Formula weight**	564.64	534.69	494.59	517.58
**Crystal habit**	Colorless needle	Clear colorless block	Colorless plate	Colorless block
**Crystal size (mm)**	0.56 x 0.05 x 0.04	0.54 x 0.33 x 0.03	0.58 x 0.44 x 0.16	0.28 x 0.08 x 0.02
**Crystal system**	Monoclinic	Monoclinic	Monoclinic	Triclinic
**Space group (no.)**	*P2₁/c* (14)	*P2₁/n* (14)	*P2₁* (4)	*P-1* (2)
***a* (Å)**	7.2690(4)	7.1139(16)	7.5296(3)	6.9708(2)
***b* (Å)**	18.0377(10)	15.034(3)	7.7105(3)	11.7529(3)
***c* (Å)**	9.8532(6)	12.418(3)	20.5349(8)	15.8221(4)
α **(°)**	90	90	90	108.4810(10)
β **(°)**	100.550(3)	91.364(12)	99.764(2)	94.4340(10)
γ **(°)**	90	90	90	92.8220(10)
V **(Å^3)**	1282.96(13)	1327.8(5)	1174.92(8)	1222.00(6)
Z	2	2	2	2
D_c **(g cm^{-3})**	1.462	1.337	1.398	1.407
***F*(000)**	592	576	524	544
μ **(mm^{-1})**	0.263	2.71	0.276	2.441
**Total reflections**	17544	6331	62164	31375
**Unique reflections**	2620	2928	11383	4949
R_{int}[d]	0.0580	n/a	0.0449	0.0238
R_1[a] **[$I > 2\sigma(I)$]**	0.0392	0.0947	0.0488	0.0268
wR_2[b] **(all data)**	0.0924	0.2590	0.1321	0.0749
**GOF (all data)**	1.079	1.189	1.126	1.054
**Flack parameter**[c]	n/a	n/a	0.046(14)	n/a
**Restraints**	0	0	3	0
**Parameters**	172	155	292	311

[a]$R_1 = \Sigma||F_o|-|F_c||/\Sigma|F_o|$; [b]$wR_2=\{\Sigma[w(F_o^2-F_c^2)^2]/\Sigma[w(F_o^2)^2]\}^{1/2}$; [c]n.a. – not applicable; [d]n/a – not applicable because the structure is twinned.

Table S2 (continued). Detailed crystallographic data.

**Compound name**	$G_2$1,5-NDS⊃aniline (synchrotron data)	$G_2$1,5-NDS⊃(*S*)-2-hexanol	$G_2$1,5-NDS⊃benzonitirile	GCHMS⊃*cis*-1,2-dimethylcyclohexane
**Structure file name**	**8S**	**9**	**10**	**11**
**X-ray lab code**	23mdw75ay	23mdw61ay	Mds088	Mds058
**CCDC no.**	2307192	2307203	2307194	2307207
**Formula by X-ray**	$C_{18}H_{27}N_7O_7S_2$	$C_{18}H_{32}N_6O_7S_2$	$C_{20}H_{20}N_7O_6S_2$	$C_{29}H_{67}N_9O_9S_3$
**Formula weight**	517.58	508.61	610.67	782.09
**Crystal habit**	Colorless needle	Colorless block	Clear colorless block	Colorless needle
**Crystal size (mm)**	0.56 x 0.03 x 0.03	0.49 x 0.34 x 0.26	0.1 x 0.06 x 0.04	0.56 x 0.09 x 0.03
**Crystal system**	Triclinic	monoclinic	Triclinic	Orthorhombic
**Space group (no.)**	*P-1* (2)	$P2_1$ (4)	*P-1* (2)	*Cmca* (64)
a **(Å)**	6.9854(5)	7.4931(7)	11.6704(4)	21.6164(6)
b **(Å)**	11.7403(9)	7.6808(7)	12.6241(4)	12.4875(4)
c **(Å)**	15.8365(13)	21.1801(19)	14.6631(5)	31.7055(10)
α **(°)**	108.4656(16)	90	87.859(3)	90
β **(°)**	94.5021(15)	99.522(5)	84.978(3)	90
γ **(°)**	92.8987(15)	90	76.173(3)	90
V **(Å^3)**	1224.18(16)	1202.18(19)	2089.33(12)	8558.4(5)
Z	2	2	3	8
D_c **(g cm^{-3})**	1.404	1.405	1.456	1.214
F**(000)**	544	540	956	3392
μ **(mm^{-1})**	0.41328	0.272	2.737	2.043
**Total reflections**	46775	82550	35138	18321
**Unique reflections**	10915	11671	8447	4292
R_{int}	0.0653	0.0564	0.0388	0.0514
R_1[a] **[$I > 2 \sigma(I)$]**	0.0476	0.0393	0.0644	0.0772
wR_2[b] **(all data)**	0.1344	0.0986	0.1792	0.2000
**GOF (all data)**	1.072	1.243	1.087	1.131
**Flack parameter**[c]	n/a	0.073(13)	n/a	n/a
**Restraints**	5	10	0	50
**Parameters**	323	325	541	273

[a]$R_1 = \Sigma||F_o|-|F_c||/\Sigma|F_o|$; [b]$wR_2=\{\Sigma[w(F_o^2-F_c^2)^2]/\Sigma[w(F_o^2)^2]\}^{1/2}$; [c]n.a. – not applicable.

Table S2 (continued). Detailed crystallographic data.

**Compound name**	$G_2$1,5-NDS⊃(*R*)-(+)-methylcyclohexanone	$G_2$1,5-NDS⊃(*S*)-1-chloro-3-methoxypropan-2-ol	GCHMS⊃(2-methylcyclohexyl) methanol	$G_2$1,5-NDS⊃acetophenone
**Structure file name**	**12**	**13**	**14**	**15**
**X-ray lab code**	23mdw26ay	23mdw52ay	23mdw19d	23mdw22ay
**CCDC no.**	2307199	2307200	2307210	2307197
**Formula by X-ray**	$C_{19}H_{30}N_6O_7S_2$	$C_{16}H_{27}ClN_6O_8S_2$	$C_{29}H_{67}N_9O_{10}S_3$	$C_{28}H_{34}N_6O_8S_2$
**Formula weight**	518.61	531.00	798.09	646.73
**Crystal habit**	Clear colorless plate	Colorless plate	Colorless needle	Colorless plate
**Crystal size (mm)**	0.56 x 0.40 x 0.18	0.47 x 0.42 x 0.09	0.33 x 0.09 x 0.06	0.56 x 0.44 x 0.16
**Crystal system**	orthorhombic	monoclinic	Monoclinic	Monoclinic
**Space group (no.)**	$P2_12_12_1$ (19)	$P2_1$ (4)	$P2_1/c$ (14)	$P2_1/n$ (14)
a **(Å)**	7.7492(2)	7.5851(3)	12.4616(6)	7.6261(3)
b **(Å)**	14.9332(4)	7.6267(3)	31.3075(14)	20.0389(9)
c **(Å)**	21.6566(6)	20.2491(8)	12.3952(6)	10.3667(5)
α **(°)**	90	90	90	90
β **(°)**	90	99.638(3)	119.285(2)	94.632(2)
γ **(°)**	90	90	90	90
V **(Å^3)**	2506.11(12)	1165.13(8)	4217.8(4)	1579.05(12)
Z	4	2	4	2
D_c **(g cm^{-3})**	1.3675	1.514	1.257	1.360
F**(000)**	1096	556	1728	680
μ **(mm^{-1})**	0.263	0.398	0.234	0.226
**Total reflections**	47126	85231	75764	25387
**Unique reflections**	7041	10695	10533	3941
R_{int}	0.0457	0.0415	0.0745	0.0448
R_1[a] $[I > 2\sigma(I)]$	0.0380	0.0349	0.0636	0.0353
wR_2[b] **(all data)**	0.0999	0.0840	0.1629	0.0915
**GOF (all data)**	1.067	1.110	1.064	1.042
**Flack parameter**[c]	0.03(2)	0.032(11)	n/a	n/a
**Restraints**	0	84	29	0
**Parameters**	308	434	549	200

[a]$R_1 = \Sigma||F_o|-|F_c||/\Sigma|F_o|$; [b]$wR_2=\{\Sigma[w(F_o^2-F_c^2)^2]/\Sigma[w(F_o^2)^2]\}^{1/2}$; [c]n.a. – not applicable.

Table S2 (continued). Detailed crystallographic data.

**Compound name**	$G_2$1,5-NDS⊃1-phenyl-1-propanol	$G_2$1,5-NDS⊃(*S*)-(-)-limonene	GCHMS⊃(*R*)-(+)-limonene	GCHMS⊃γ-terpinene
**Structure file name**	**16**	**17**	**18**	**19**
**X-ray lab code**	Mds059 23mdw9ay	Mds389	AY129F	ay138
**CCDC no.**	2307205	2312929	2308623	2307214
**Formula by X-ray**	$C_{21}H_{30}N_6O_7S_2$	$C_{11}H_{17}N_3O_3S$	$C_{36.49}H_{81.58}N_{12}O_{12}S_4$	$C_{38}H_{84}N_{12}O_{12}S_4$
**Formula weight**	542.63	271.33	1008.77	1029.41
**Crystal habit**	Colorless plate	Colorless block	Colorless plate	Clear colorless needle
**Crystal size (mm)**	0.29 x 0.22 x 0.02	0.21 x 0.13 x 0.07	0.22 x 0.17 x 0.02	0.46 x 0.02 x 0.01
**Crystal system**	Monoclinic	Orthorhombic	Triclinic	Monoclinic
**Space group (no.)**	$P2_1/n$ (14)	$P2_12_12$ (18)	*P1* (1)	*Pc* (7)
a **(Å)**	7.5237(2)	21.2475(8)	8.5832(4)	12.1982(5)
b **(Å)**	21.2478(7)	8.1618(3)	17.6798(7)	14.1654(6)
c **(Å)**	7.9048(3)	7.5487(2)	19.0779(7)	16.5069(7)
α **(°)**	90	90	81.553(3)	90
β **(°)**	95.059(3)	90	89.200(3)	90.010(3)
γ **(°)**	90	90	89.848(3)	90
V **(Å^3)**	1258.76(7)	1309.07(8)	2863.4(2)	2852.3(2)
Z	2	4	2	2
D_c **(g cm^{-3})**	1.432	1.377	1.170	1.199
F**(000)**	572	576	1089	1112
μ **(mm^{-1})**	2.385	2.261	2.021	2.039
**Total reflections**	11735	41102	153676	44919
**Unique reflections**	2504	2339	22086	9026
R_{int}	0.0420	0.0567	0.0694	0.1431
R_1^a **[$I > 2\sigma(I)$]**	0.0536	0.0507	0.0644	0.1453
wR_2^b **(all data)**	0.1430	0.1492	0.1798	0.3134
**GOF (all data)**	1.068	1.006	1.042	1.256
**Flack parameter^c**	n/a	0.12(6)	-0.062(13)	0.135(18)
**Restraints**	69	138	1476	916
**Parameters**	228	199	1428	611

$^aR_1 = \Sigma||F_o|-|F_c||/\Sigma|F_o|$; $^bwR_2=\{\Sigma[w(F_o^2-F_c^2)^2]/\Sigma[w(F_o^2)^2]\}^{1/2}$; ^{c}n.a. – not applicable.

Table S2 (continued). Detailed crystallographic data.

**Compound name**	G$_2$1,5-NDS⊃(*R*)-(-)-carvone	G$_2$1,5-NDS⊃(*S*)-(+)-carvone	G$_2$1,5-NDS⊃methyl-L-pyroglutamate	GCHMS⊃(-)-α-thujone[d]
**Structure file name**	**20**	**21**	**22**	**23**
**X-ray lab code**	23mdw95ay	23mdw94ay	23mdw40ay	23mdw69ay
**CCDC no.**	2307190	2307189	2307198	2307213
**Formula by X-ray**	$C_{22}H_{32}N_6O_7S_2$	$C_{22}H_{32}N_6O_7S_2$	$C_{18}H_{27}N_7O_9S_2$	$C_{66}H_{152}N_{24}O_{25}S_8$
**Formula weight**	556.65	556.65	549.58	1938.59
**Crystal habit**	Clear colorless block	Clear colorless block	Clear colorless block	Clear colorless plate
**Crystal size (mm)**	0.32 x 0.18 x 0.02	0.32 x 0.18 x 0.02	0.23 x 0.18 x 0.08	0.22 x 0.12 x 0.01
**Crystal system**	Orthorhombic	Orthorhombic	Triclinic	Triclinic
**Space group (no.)**	$P2_12_12_1$ (19)	$P2_12_12_1$ (19)	*P1* (1)	*P1* (1)
a **(Å)**	8.3277(3)	8.3418(2)	7.5128(3)	8.6135(6)
b **(Å)**	14.8298(5)	14.8262(4)	8.2137(4)	17.5833(12)
c **(Å)**	21.3403(8)	21.3126(5)	9.6058(4)	19.1134(12)
α **(°)**	90	90	88.1062(14)	80.851(5)
β **(°)**	90	90	86.4041(14)	89.256(5)
γ **(°)**	90	90	89.8790(14)	89.920(5)
V **(Å^3)**	2635.49(16)	2635.88(11)	591.26(4)	2857.7(3)
Z	4	4	1	1
D_c **(g cm^{-3})**	1.403	1.403	1.543	1.126
F**(000)**	1176	1176	288	1044
**μ (mm^{-1})**	2.293	2.292	0.290	2.014
**Total reflections**	85568	68430	17594	100701
**Unique reflections**	5374	5399	5573	19437
R_{int}	0.0465	0.0340	0.0259	0.1010
R_1[a] **[*I* > 2 ʃ(*I*)]**	0.0419	0.0268	0.0286	0.1382
wR_2[b] **(all data)**	0.1141	0.0747	0.0737	0.3514
**GOF (all data)**	1.109	1.048	1.072	1.125
**Flack parameter**[c]	0.006(5)	-0.006(3)	0.057(18)	-0.05(4)
**Restraints**	0	0	4	1015
**Parameters**	336	336	330	1139

[a] $R_1 = \Sigma||F_o|-|F_c||/\Sigma|F_o|$; [b] $wR_2=\{\Sigma[w(F_o^2-F_c^2)^2]/\Sigma[w(F_o^2)^2]\}^{1/2}$; [c] n.a. – not applicable; [d] Molecular formula reflects a solvent masked structure wherein one thujone molecule has been removed due to disorder.

Table S2 (continued). Detailed crystallographic data.

**Compound name**	GCHMS⊃*cis*-rose oxide	GCHMS⊃*cis*-rose oxide (synchrotron data)	GCHMS⊃eucalyptol	GCHMS⊃geraniol [d]
**Structure file name**	**24**	**24S**	**25**	**26**
**X-ray lab code**	23mdw67ay	23mdw73ay	23mdw110ay	AY129-G
**CCDC no.**	2307217	2307208	2307212	2307218
**Formula by X-ray**	$C_{38}H_{84}N_{12}O_{13}S_4$	$C_{38}H_{86}N_{12}O_{13}S_4$	$C_{17}H_{35}N_3O_4S$	$C_{19}H_{43}N_6O_{6.5}S_2$
**Formula weight**	1045.41	1046.42	377.54	523.71
**Crystal habit**	Clear colorless plate	Colorless plate	Clear colorless plate	Colorless plate
**Crystal size (mm)**	0.51 x 0.12 x 0.02	0.51 x 0.12 x 0.02	0.44 x 0.12 x 0.01	0.36 x 0.19 x 0.02
**Crystal system**	Orthorhombic	Orthorhombic	Monoclinic	Monoclinic
**Space group (no.)**	$Pca2_1$ (29)	$Pca2_1$ (29)	$P2_1/n$ (14)	$P2_1/c$ (14)
a **(Å)**	28.4245(6)	28.480(2)	7.0137(7)	12.3748(6)
b **(Å)**	8.4862(2)	8.4230(7)	11.1893(12)	15.9156(7)
c **(Å)**	23.8945(5)	23.9364(19)	27.288(3)	14.4666(7)
α **(°)**	90	90	90	90
β **(°)**	90	90	90.252(7)	90.821(2)
γ **(°)**	90	90	90	90
V **(Å^3)**	5763.7(2)	5742.0(8)	2141.5(4)	2848.9(2)
Z	4	4	4	4
D_c **(g cm^{-3})**	1.205	1.212	1.171	1.221
F**(000)**	2256	2264	824	1132
μ **(mm^{-1})**	0.064	0.064	1.542	2.063
**Total reflections**	125128	219235	25647	61315
**Unique reflections**	11725	27186	3282	5137
R_{int}	0.0777	0.0876	0.0885	0.0686
R_1[a] $[I > 2\sigma(I)]$	0.0679	0.0450	0.1135	0.0454
wR_2[b] **(all data)**	0.1736	0.1143	0.2690	0.1298
**GOF (all data)**	1.043	1.079	1.230	1.043
**Flack parameter**[c]	-0.01(2)	0.12(6)	n/a	n/a
**Restraints**	512	4	596	231
**Parameters**	740	707	331	350

[a] $R_1 = \Sigma||F_o|-|F_c||/\Sigma|F_o|$; [b] $wR_2=\{\Sigma[w(F_o^2-F_c^2)^2]/\Sigma[w(F_o^2)^2]\}^{1/2}$; [c] n.a. – not applicable, [d] The geraniol guest is disordered about an inversion center in the void. Therefore, the refinement used a model wherein one of the 50% occupancy guests was removed, as reflected in the moiety formula.

Table S2 (continued). Detailed crystallographic data.

**Compound name**	$G_2$1,5-NDS⊃(*R*)-(-)-2-chloro-1-phenylethanol	GCHMS⊃eugenol [d]	$G_2$1,5-NDS⊃eugenol[d]	GCHMS⊃nicotine
**Structure file name**	**27**	**28a**	**28b**	**29**
**X-ray lab code**	23mdw101ay	23mdw79ay	Mds352	23mdw80ay
**CCDC no.**	2307195	2307216	2310649	2307209
**Formula by X-ray**	$C_{100}H_{135}Cl_{15}N_{30}O_{35}S_{10}$	$C_{14}H_{34}N_6O_{6.5}S_2$	$C_{11}H_{15}N_3O_4S$	$C_{38}H_{82}N_{14}O_{12}S_4$
**Formula weight**	2815.22	446.59	285.32	1055.4
**Crystal habit**	Clear colorless block	Clear colorless block	Dull colorless irregular	Colorless plate
**Crystal size (mm)**	0.99 x 0.51 x 0.31	0.31 x 0.05 x 0.02	0.34 x 0.22 x 0.17	0.36 x 0.22 x 0.02
**Crystal system**	Orthorhombic	Monoclinic	Orthorhombic	Triclinic
**Space group (no.)**	$P2_12_12_1$ (19)	*Cc* (9)	*Pnnm* (58)	*P1* (1)
a **(Å)**	14.9769(3)	21.3686(15)	21.2095(8)	8.58540(10)
b **(Å)**	21.3357(5)	13.8975(10)	8.3261(3)	17.5870(2)
c **(Å)**	39.6415(8)	12.2639(8)	7.5872(3)	19.2061(3)
α **(°)**	90	90	90	98.9960(10)
β **(°)**	90	124.914(4)	90	90.0870(10)
γ **(°)**	90	90	90	91.6470(10)
V **(Å^3)**	12667.1(5)	2986.5(4)	1339.84(9)	2863.05(7)
Z	4	4	4	2
D_c **(g cm^{-3})**	1.476	0.993	1.414	1.224
***F*(000)**	5880	960	600	1136
μ **(mm^{-1})**	1.476	1.886	2.300	2.055
**Total reflections**	316282	27675	40431	55115
**Unique reflections**	25829	4719	1533	19286
R_{int}	0.0449	0.0883	0.0681	0.0237
R_1[a] **[$I > 2\sigma(I)$]**	0.0497	0.0655	0.0553	0.0271
wR_2[b] **(all data)**	0.1418	0.1830	0.1938	0.0746
**GOF (all data)**	1.070	1.142	1.181	1.066
**Flack parameter**[c]	0.0368(18)	0.38(4)	n/a	0.020(3)
**Restraints**	1267	349	0	20
**Parameters**	1676	358	73	1337

[a]$R_1 = \Sigma||F_o|-|F_c||/\Sigma|F_o|$; [b]$wR_2=\{\Sigma[w(F_o^2-F_c^2)^2]/\Sigma[w(F_o^2)^2]\}^{1/2}$; [c]n.a. – not applicable; [d]Molecular formula reflects a solvent masked structure wherein the eugenol molecules have been removed due to disorder.

Table S2 (continued). Detailed crystallographic data.

**Compound name**	GCHMS⊃12-crown-4	GCHMS⊃15-crown-5	GCHMS⊃ROY
**Structure file name**	**30**	**31**	**32**
**X-ray lab code**	23mdw93ay	Mds190	23mdw35d
**CCDC no.**	2307215	2307196	2307193
**Formula by X-ray**	$C_{22}H_{50}N_6O_{10}S_2$	$C_{34}H_{74}N_6O_{16}S_2$	$C_{19}H_{26}N_6O_5S_2$
**Formula weight**	622.80	887.11	482.58
**Crystal habit**	Clear colorless hexagon	Clear colorless plate	Orange plate
**Crystal size (mm)**	0.42 x 0.33 x 0.16	0.37 x 0.17 x 0.09	0.58 x 0.16 x 0.02
**Crystal system**	Monoclinic	Monoclinic	Orthorhombic
**Space group (no.)**	$P2_1/n$ (14)	$P2_1/c$ (14)	*Pnma* (62)
a **(Å)**	7.1579(14)	19.9930(2)	26.0394(12)
b **(Å)**	17.353(4)	15.8102(2)	6.9906(3)
c **(Å)**	12.466(3)	14.62810(10)	12.8066(6)
α **(°)**	90	90	90
β **(°)**	90.084(6)	97.0360(10)	90
γ **(°)**	90	90	90
V **(Å^3)**	1548.4(5)	4589.02(8)	2331.20(18)
Z	2	4	4
D_c **(g cm^{-3})**	1.336	1.284	1.375
F**(000)**	672	1920	1016
**μ (mm^{-1})**	0.231	1.652	2.440
**Total reflections**	28321	43902	37002
**Unique reflections**	3872	9103	2568
R_{int}	0.0617	0.0357	0.0743
R_1[a] **[$I > 2\sigma(I)$]**	0.0611	0.0559	0.1133
wR_2[b] **(all data)**	0.1521	0.1560	0.2431
**GOF (all data)**	1.136	1.029	1.416
**Flack parameter**[c]	n/a	n/a	n/a
**Restraints**	0	0	225
**Parameters**	201	523	206

[a]$R_1 = \Sigma||F_o|-|F_c||/\Sigma|F_o|$; [b]$wR_2=\{\Sigma[w(F_o^2-F_c^2)^2]/\Sigma[w(F_o^2)^2]\}^{1/2}$; [c]n.a. – not applicable.

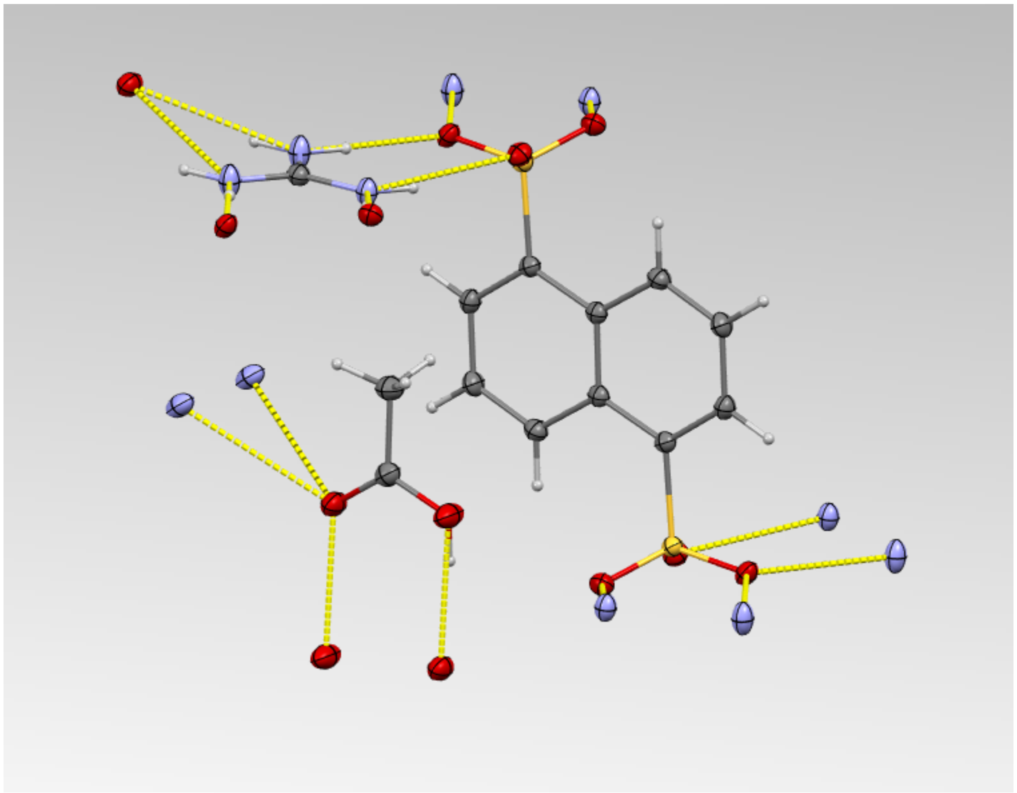

Figure S1. Molecular structure of **1** depicted as ellipsoids with 50% probability. Disordered component removed for clarity.

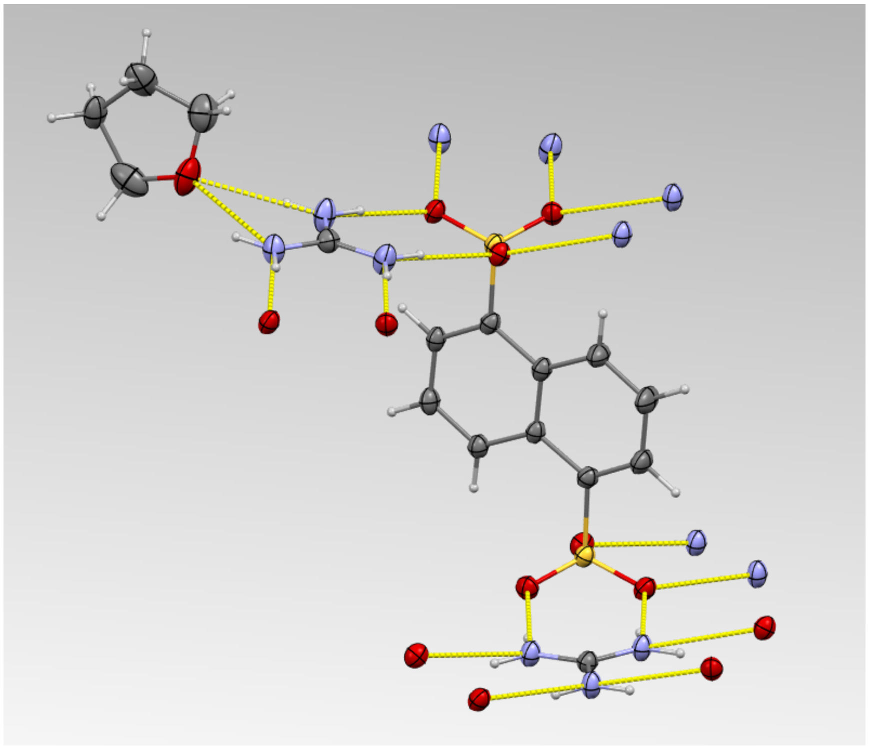

Figure S2. Molecular structure of **2** depicted as ellipsoids with 50% probability.

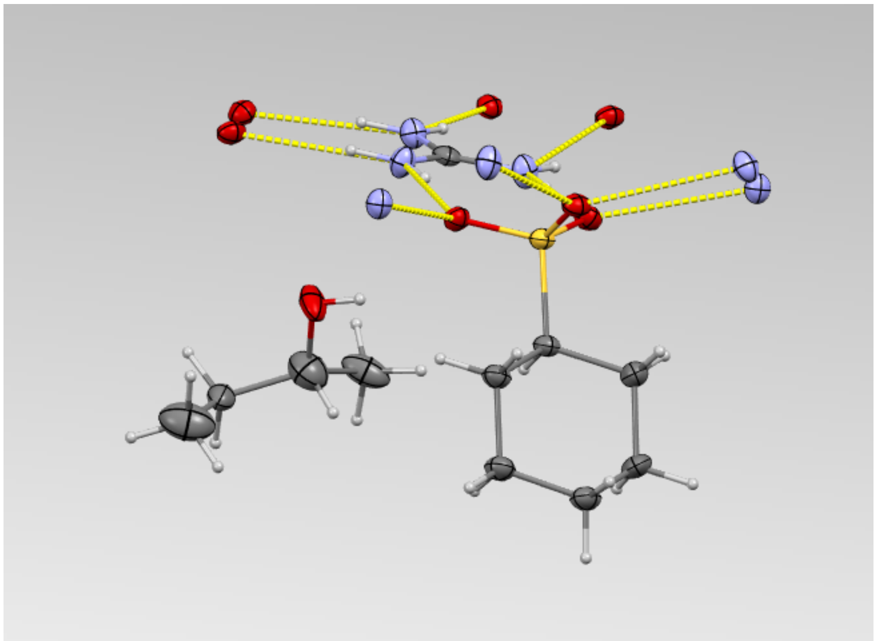

Figure S3. Molecular structure of **3** depicted as ellipsoids with 50% probability. Disordered component removed for clarity.

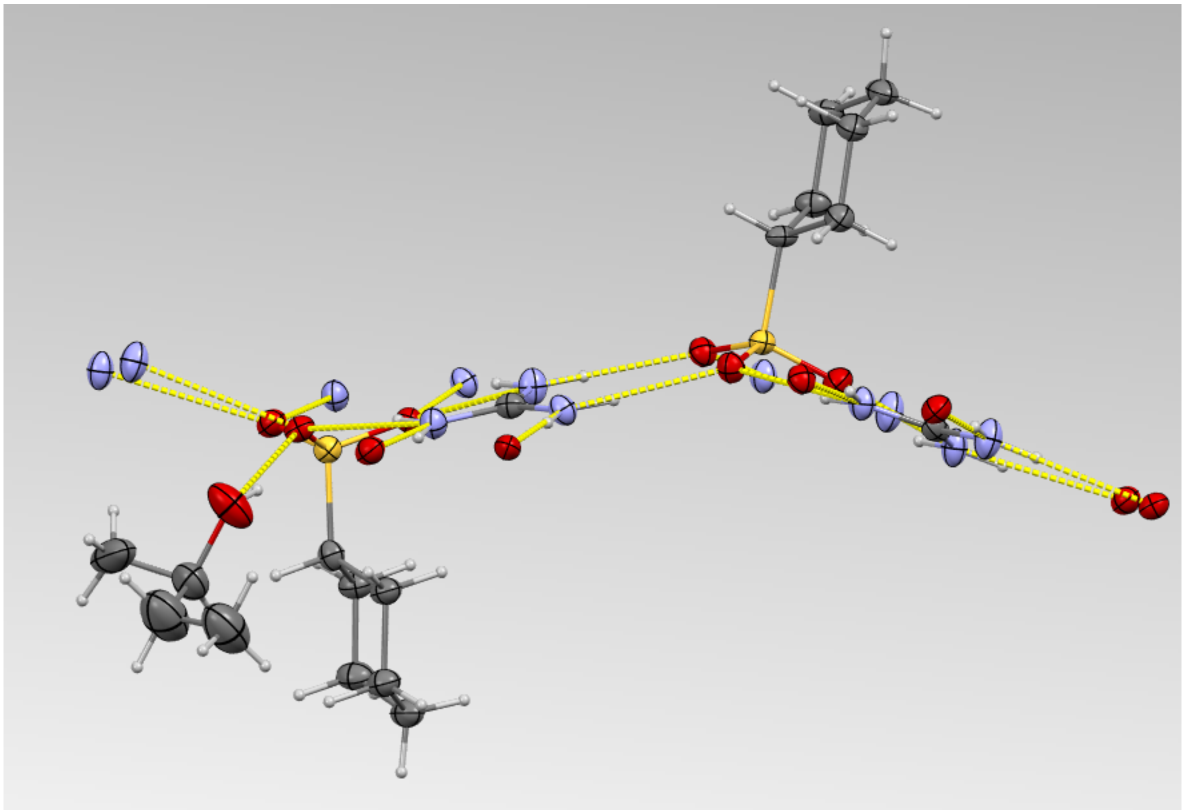

Figure S4. Molecular structure of **4** depicted as ellipsoids with 50% probability. Disordered component removed for clarity.

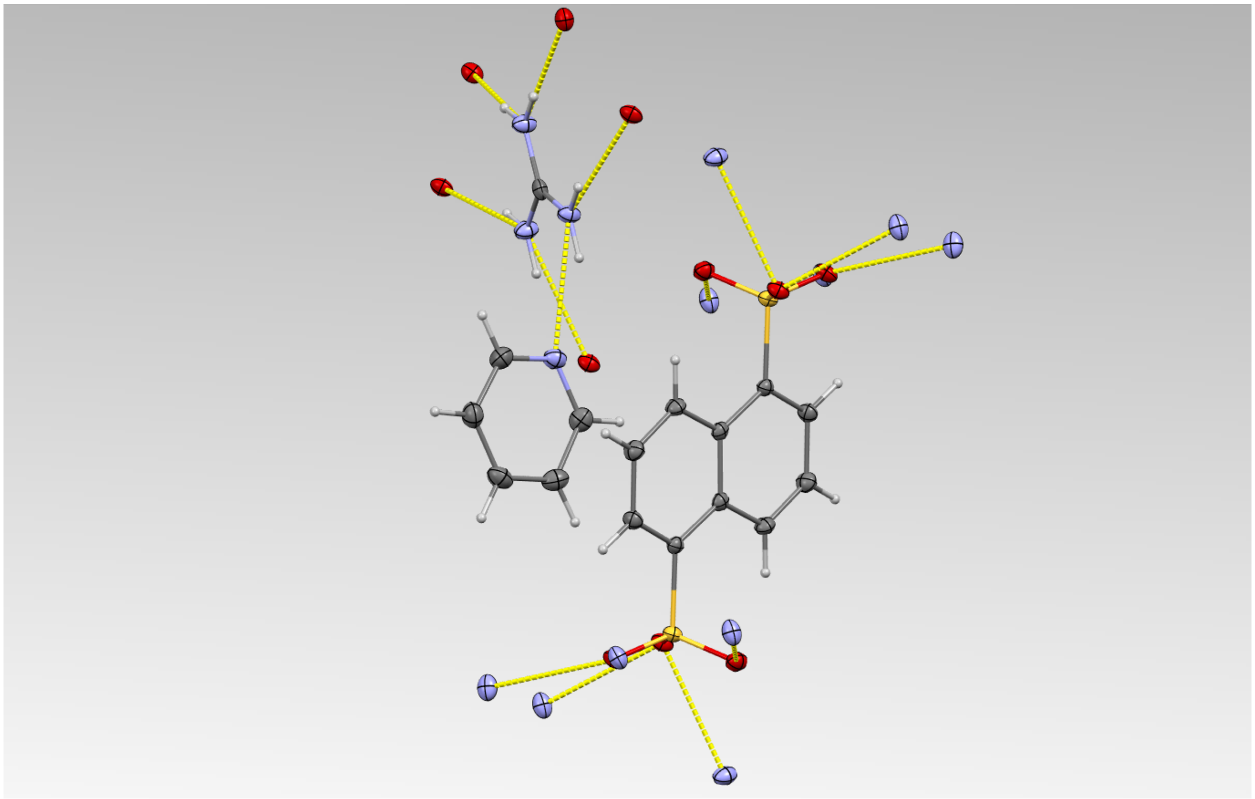

Figure S5. Molecular structure of **5** depicted as ellipsoids with 50% probability.

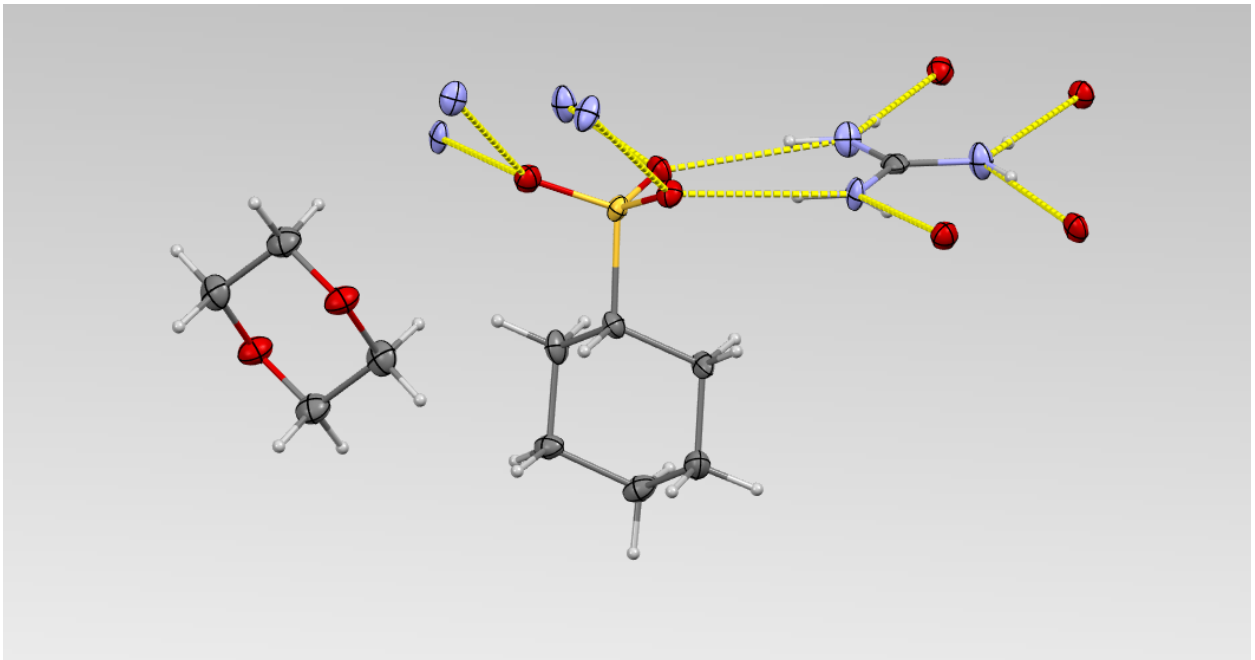

Figure S6. Molecular structure of **6** depicted as ellipsoids with 50% probability.

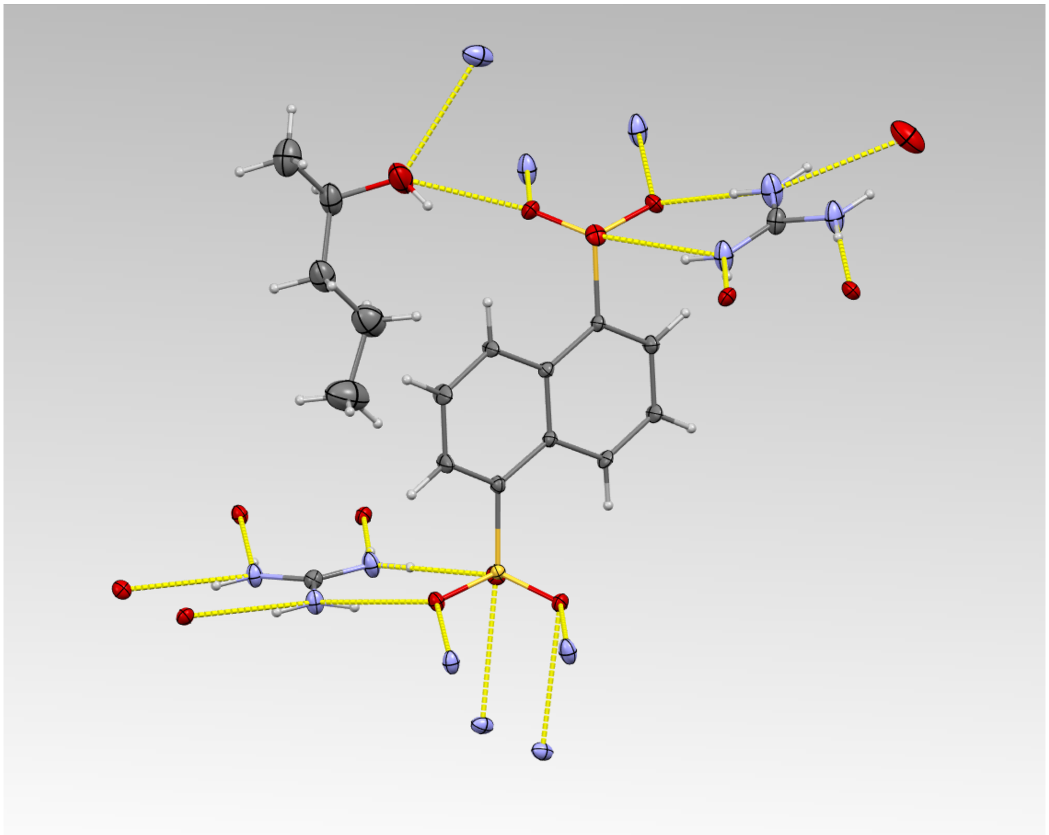

Figure S7. Molecular structure of **7** depicted as ellipsoids with 50% probability.

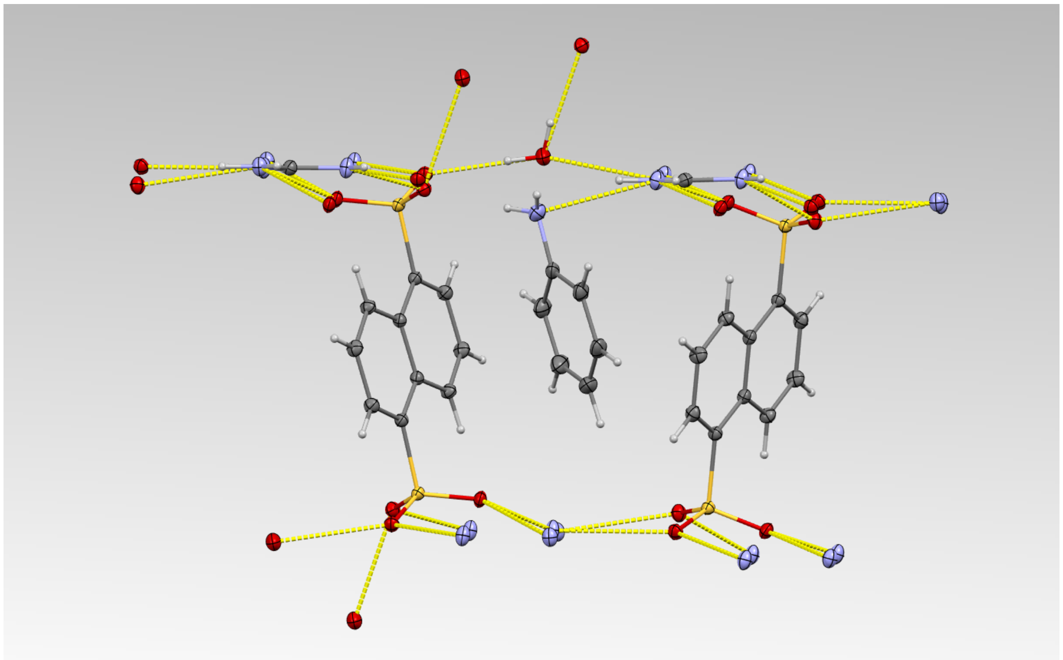

Figure S8S. Molecular structure of **8S** depicted as ellipsoids with 50% probability.

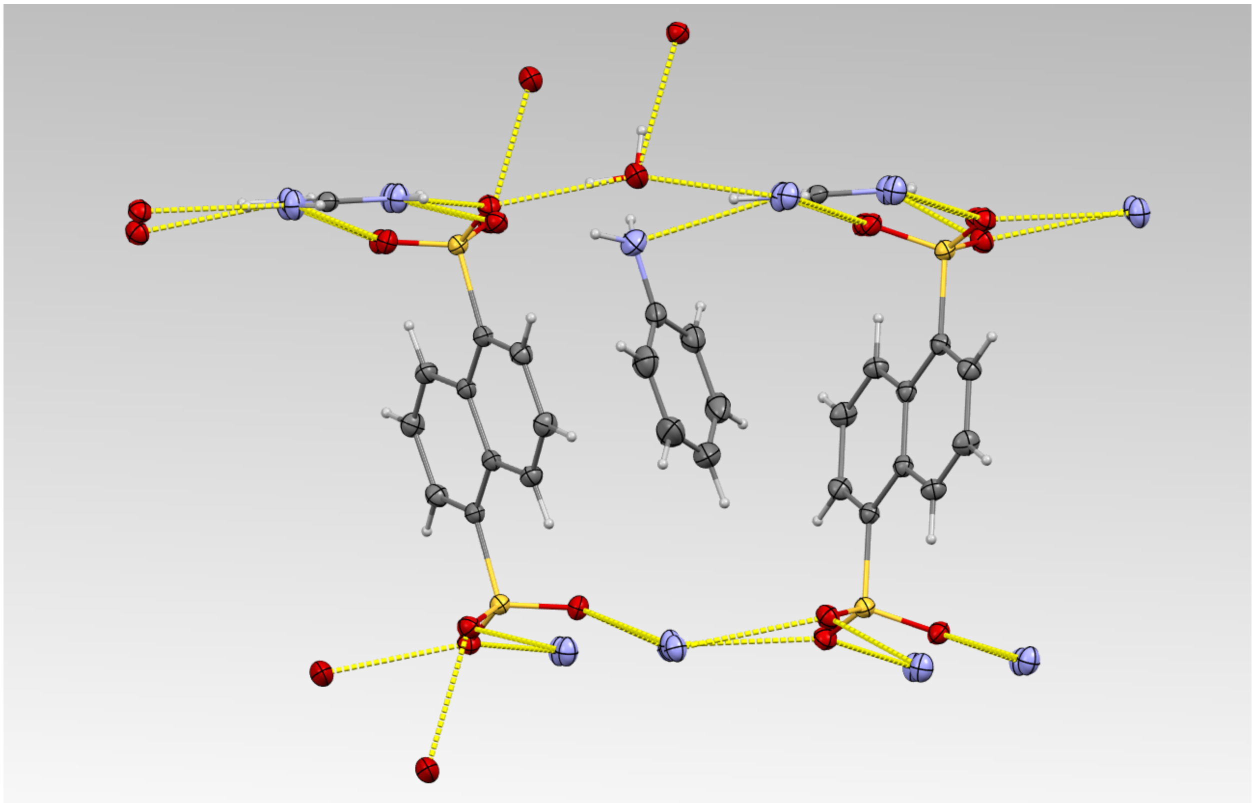

Figure S8. Molecular structure of **8** depicted as ellipsoids with 50% probability.

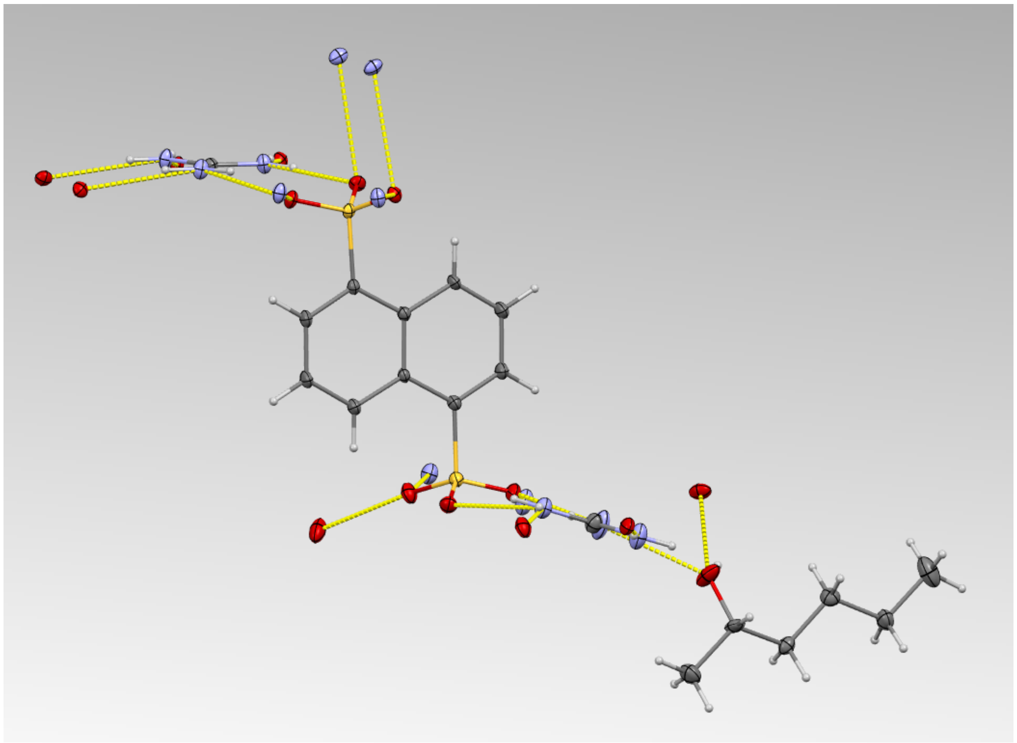

Figure S9. Molecular structure of **9** depicted as ellipsoids with 50% probability. Disordered component removed for clarity.

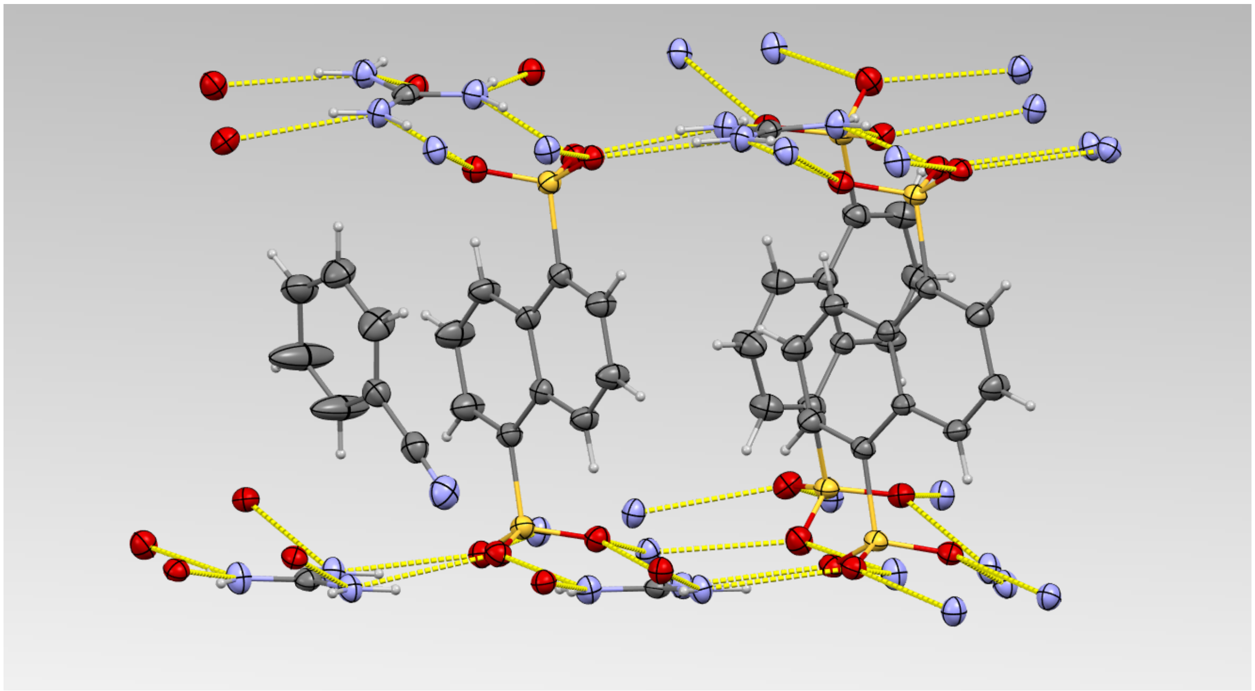

Figure S10. Molecular structure of **10** depicted as ellipsoids with 50% probability.

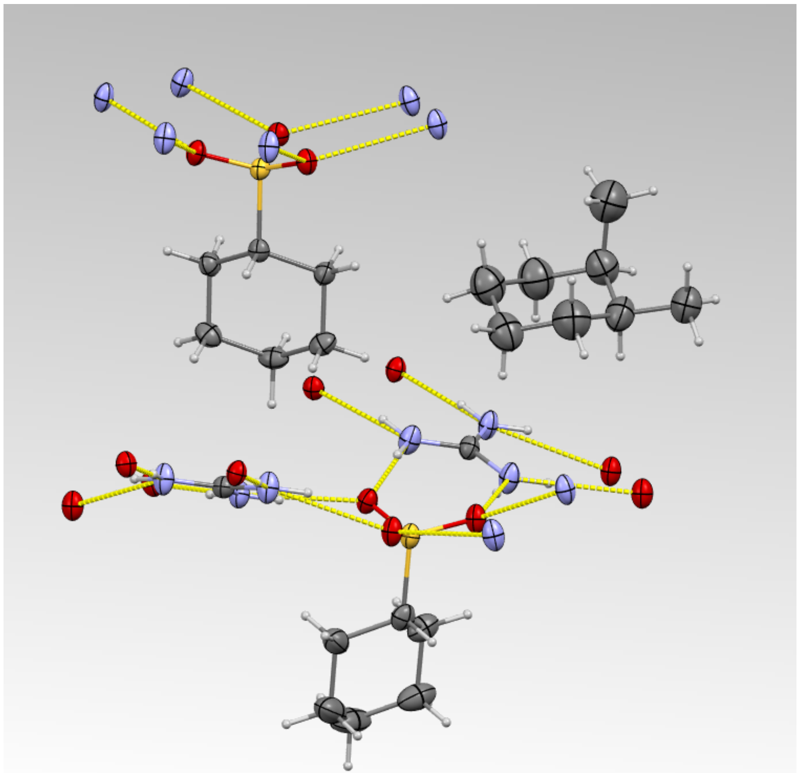

Figure S11. Molecular structure of **11** depicted as ellipsoids with 50% probability. Disordered component removed for clarity.

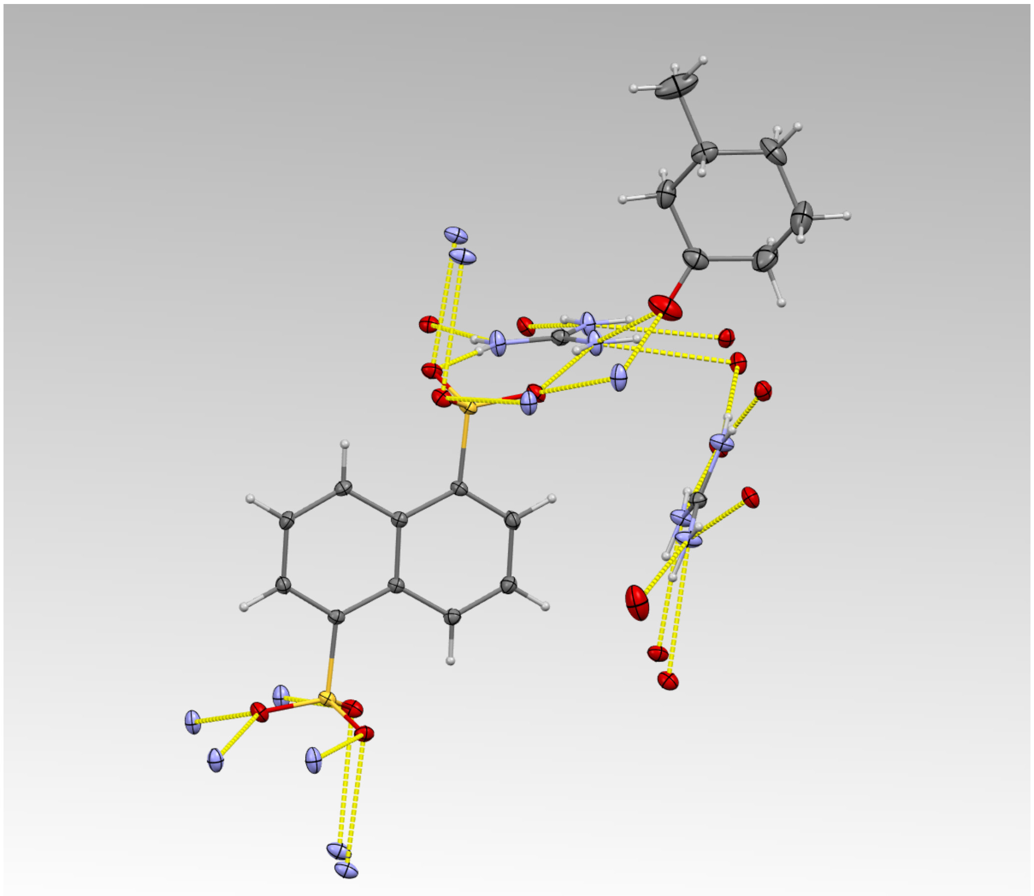

Figure S12. Molecular structure of **12** depicted as ellipsoids with 50% probability.

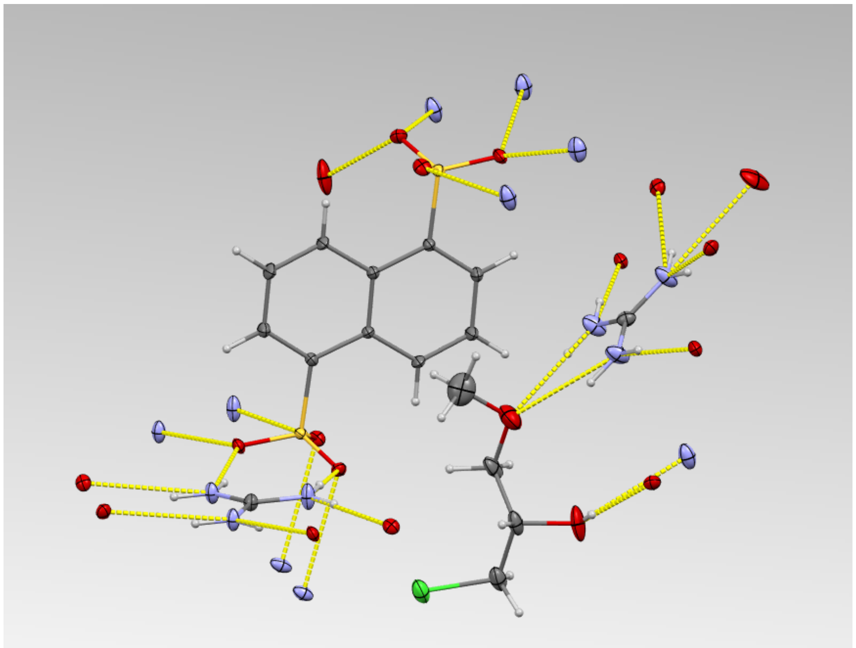

Figure S13. Molecular structure of **13** depicted as ellipsoids with 50% probability. Disordered component removed for clarity.

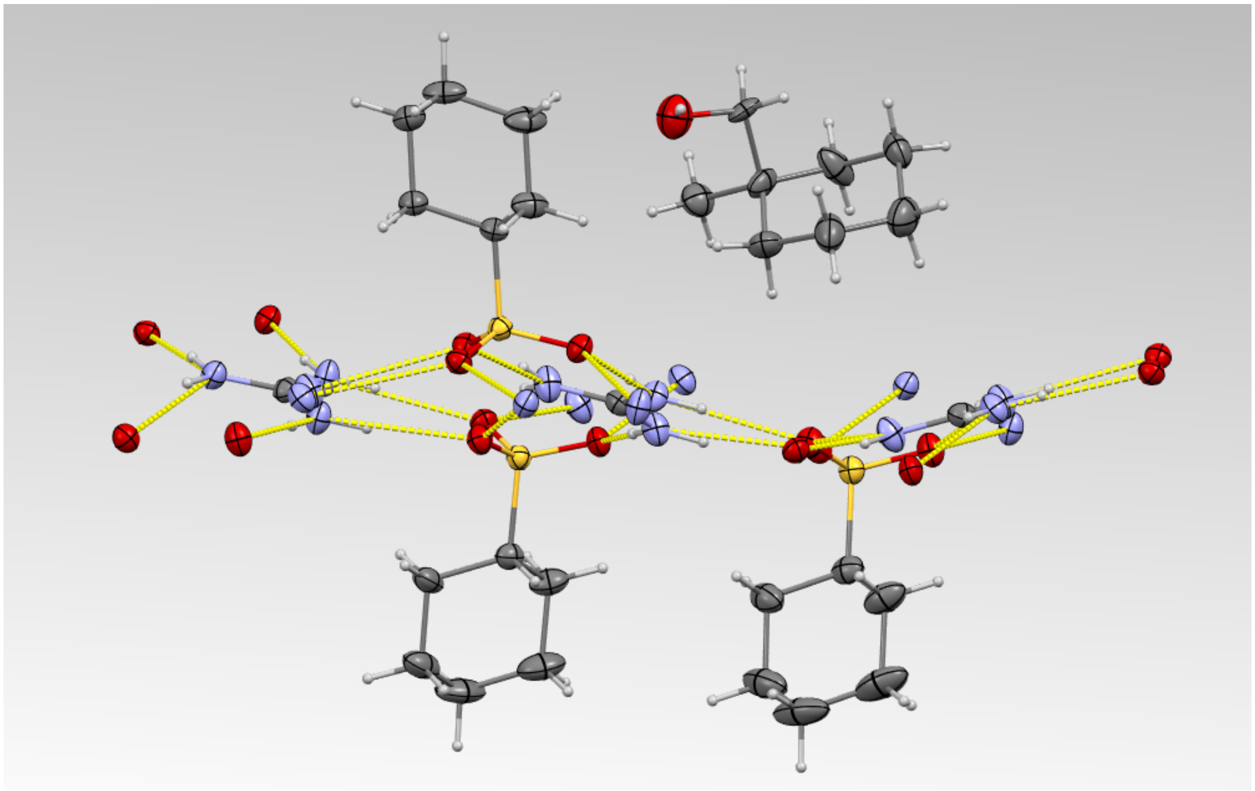

Figure S14. Molecular structure of **14** depicted as ellipsoids with 50% probability. Disordered component removed for clarity.

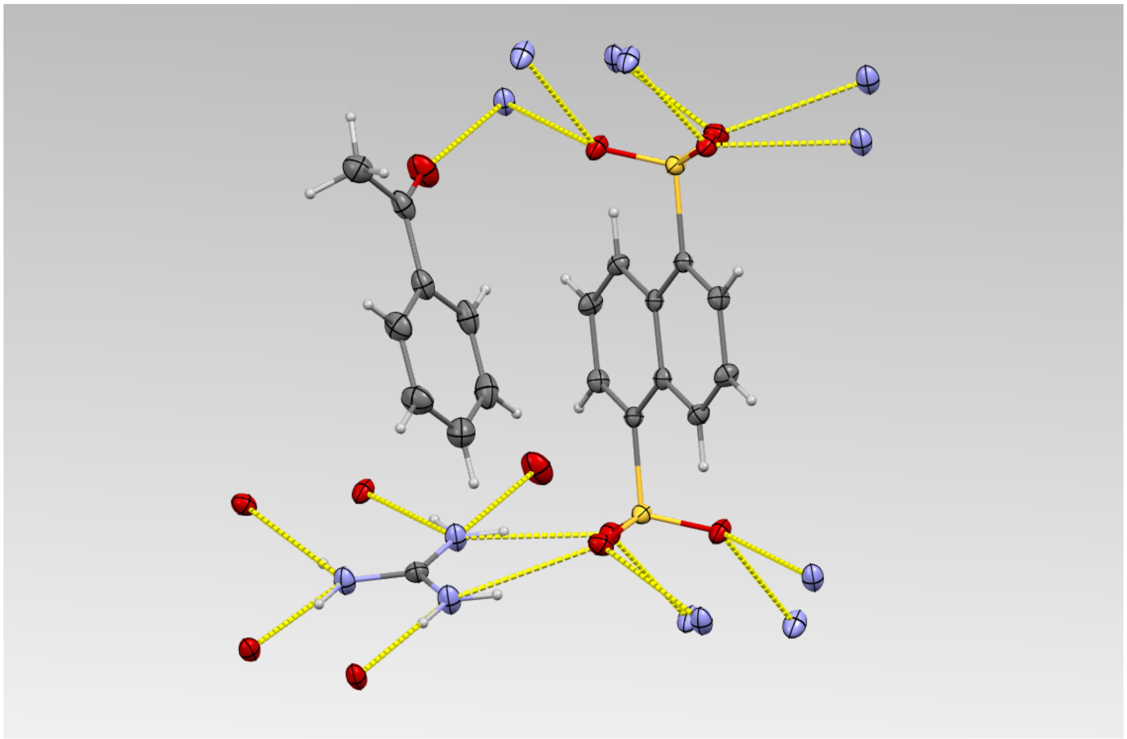

Figure S15. Molecular structure of **15** depicted as ellipsoids with 50% probability.

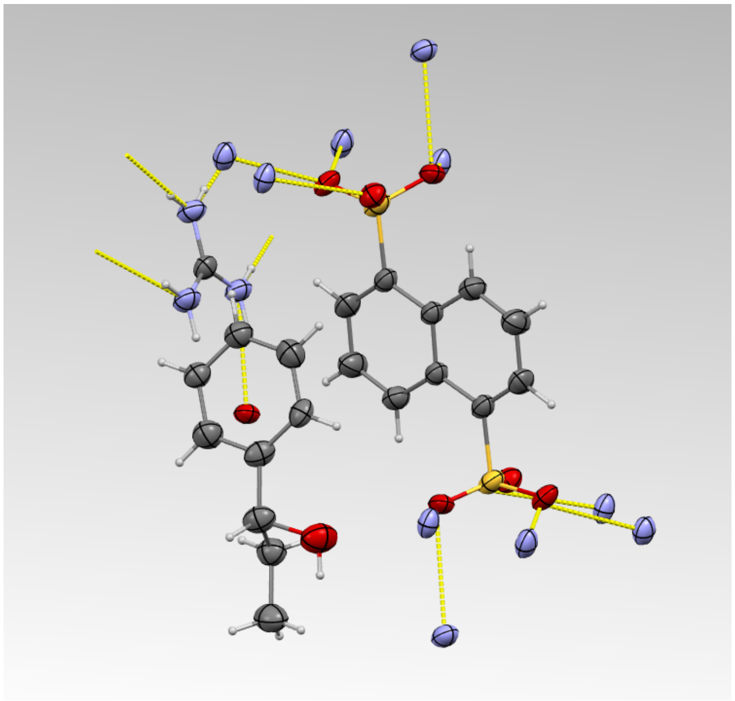

Figure S16. Molecular structure of **16** depicted as ellipsoids with 50% probability. Disordered component removed for clarity.

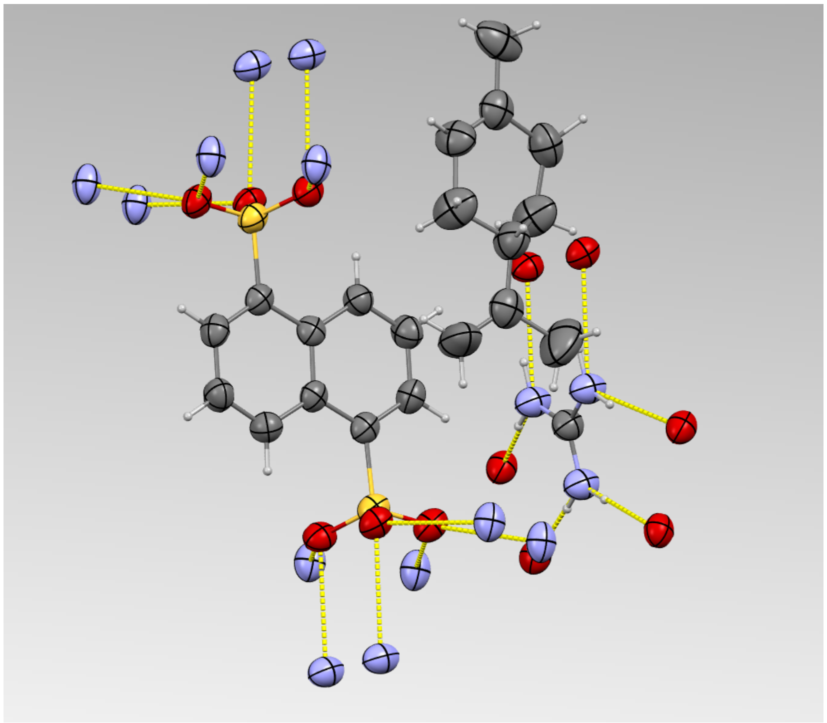

Figure S17. Molecular structure of **17** depicted as ellipsoids with 50% probability. Disordered component removed for clarity.

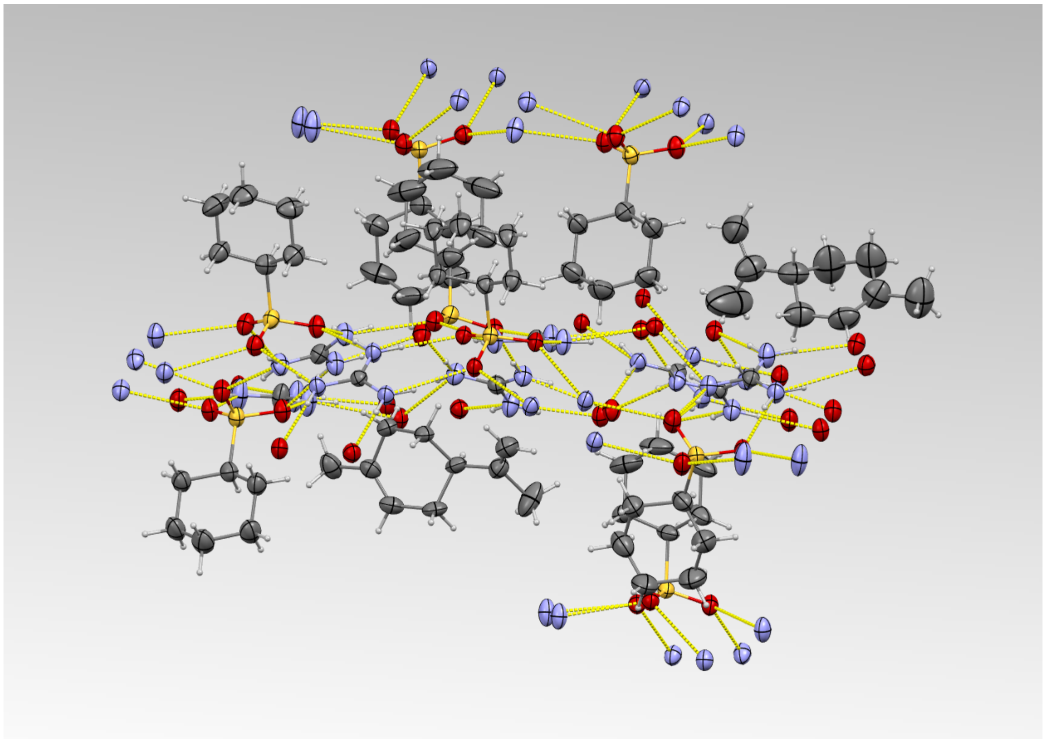

Figure S18. Molecular structure of **18** depicted as ellipsoids with 50% probability. Disordered component removed for clarity.

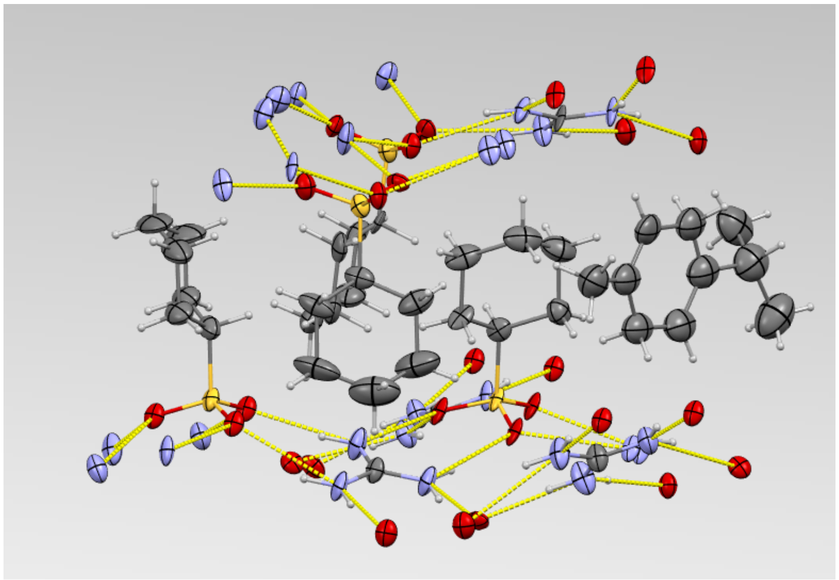

Figure S19. Molecular structure of **19** depicted as ellipsoids with 50% probability. Disordered component removed for clarity.

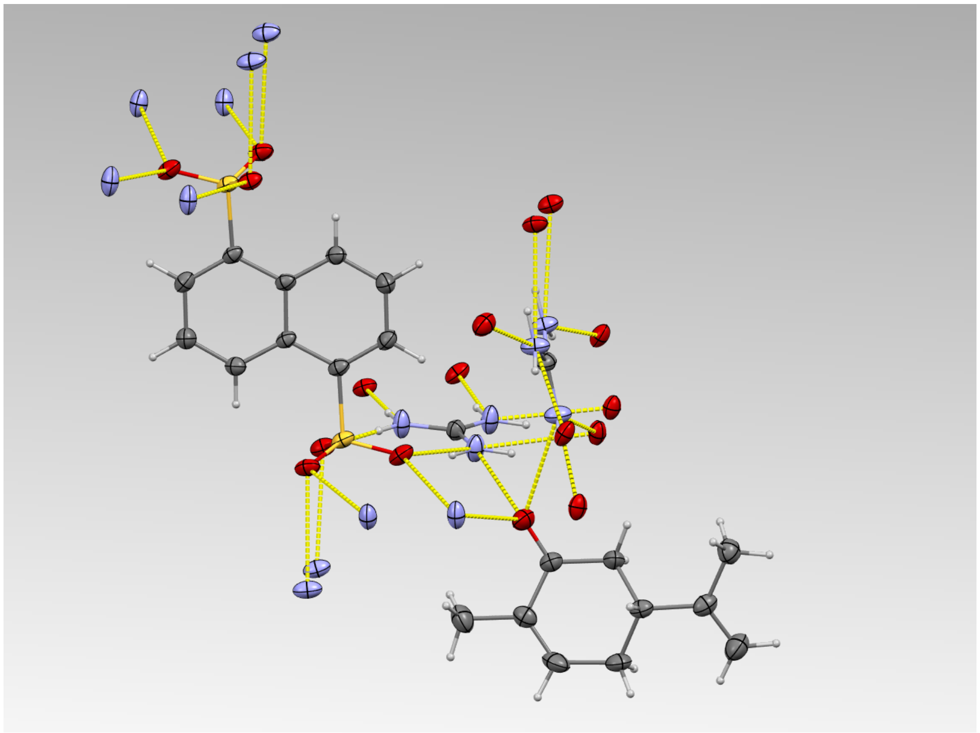

Figure S20. Molecular structure of **20** depicted as ellipsoids with 50% probability.

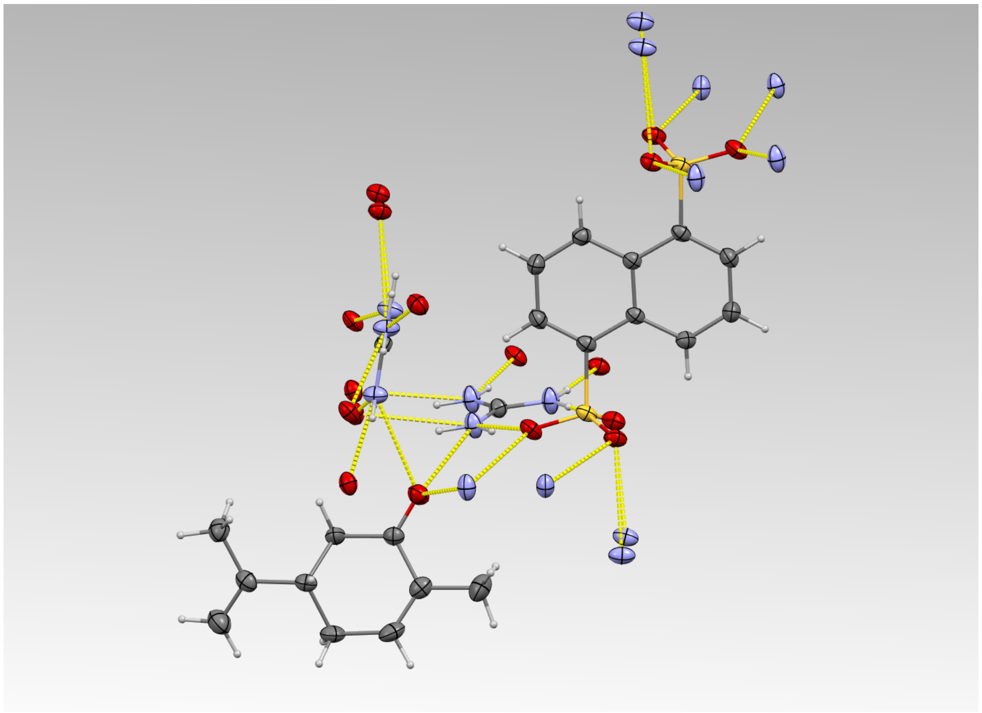

Figure S21. Molecular structure of **21** depicted as ellipsoids with 50% probability.

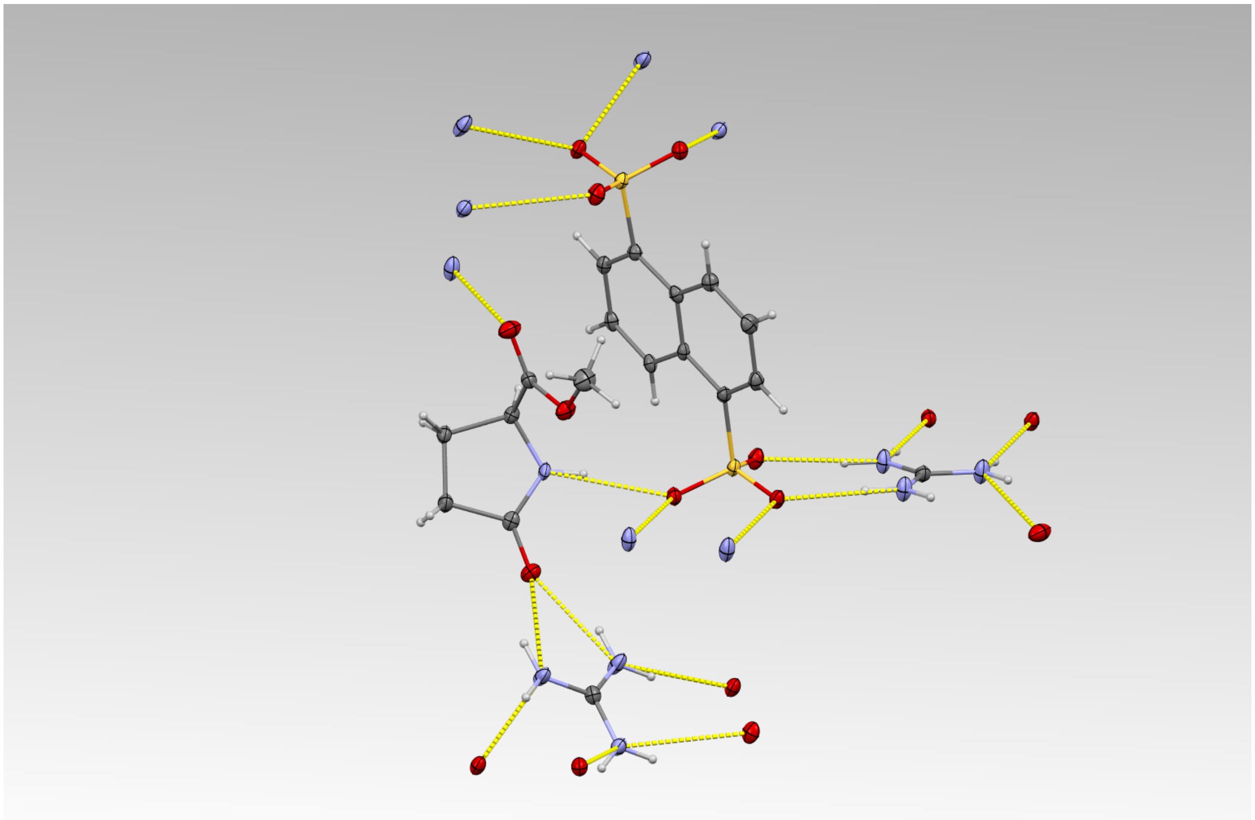

Figure S22. Molecular structure of **22** depicted as ellipsoids with 50% probability.

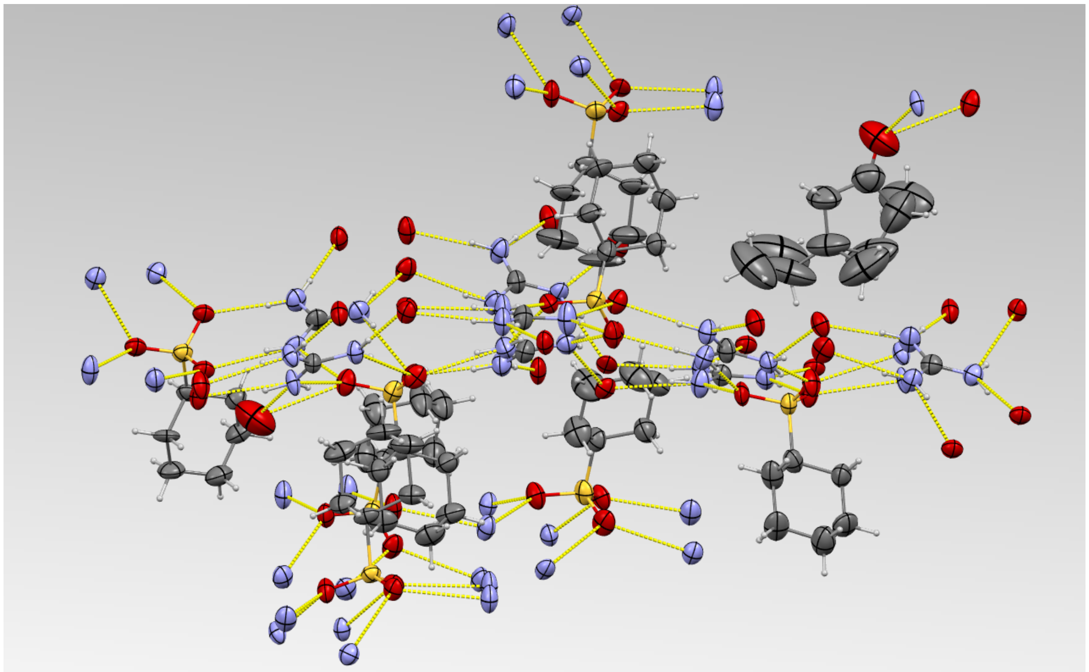

Figure S23. Molecular structure of **23** depicted as ellipsoids with 50% probability. Disordered component removed for clarity.

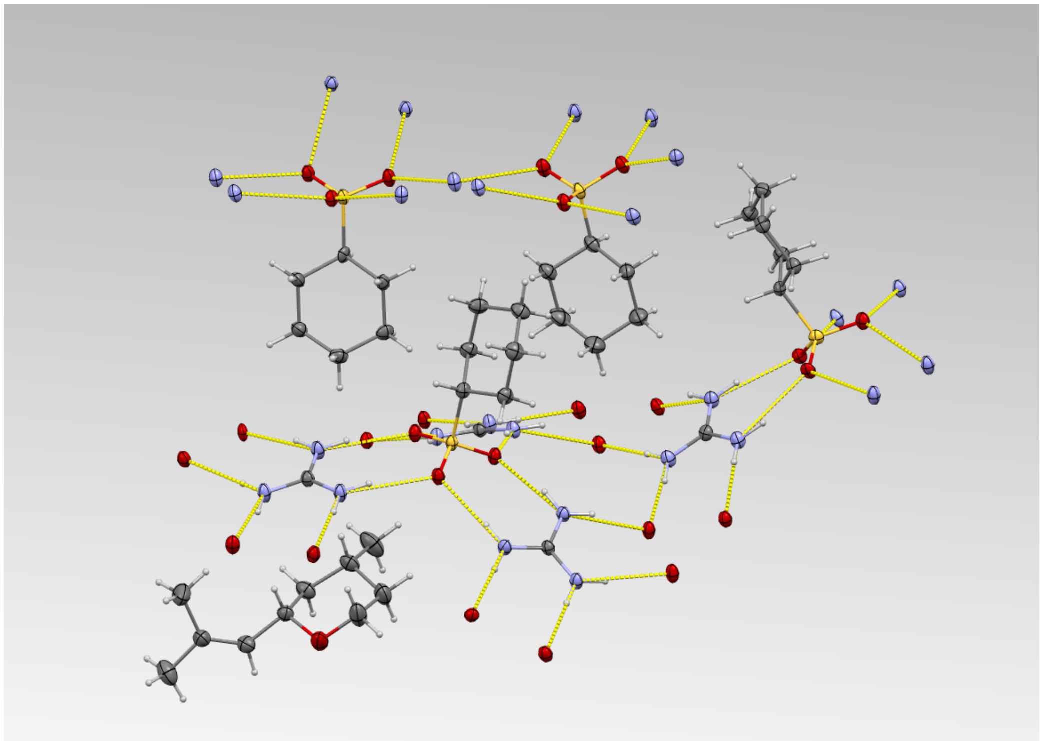

Figure S24S. Molecular structure of **24S** depicted as ellipsoids with 50% probability. Disordered component removed for clarity.

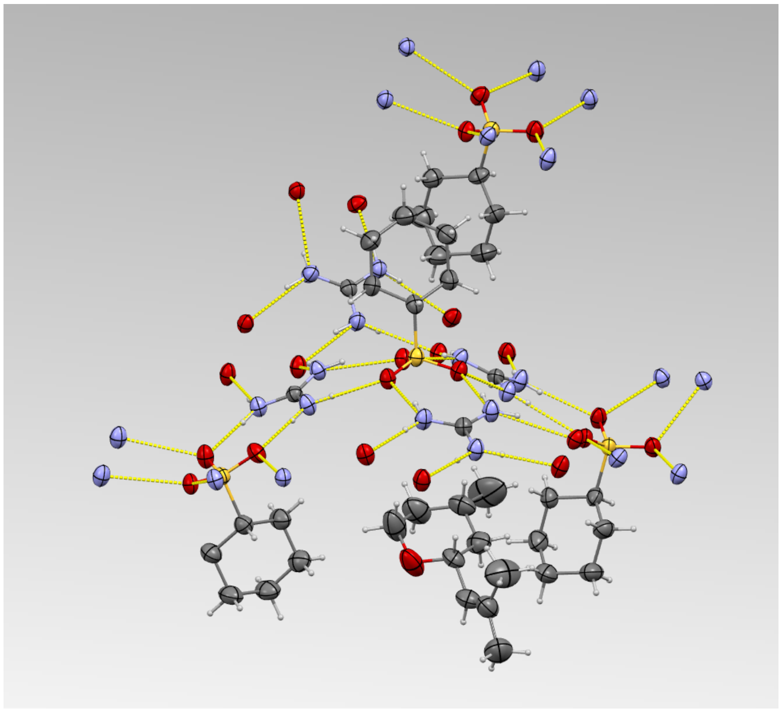

Figure S24. Molecular structure of **24** depicted as ellipsoids with 50% probability. Disordered component removed for clarity.

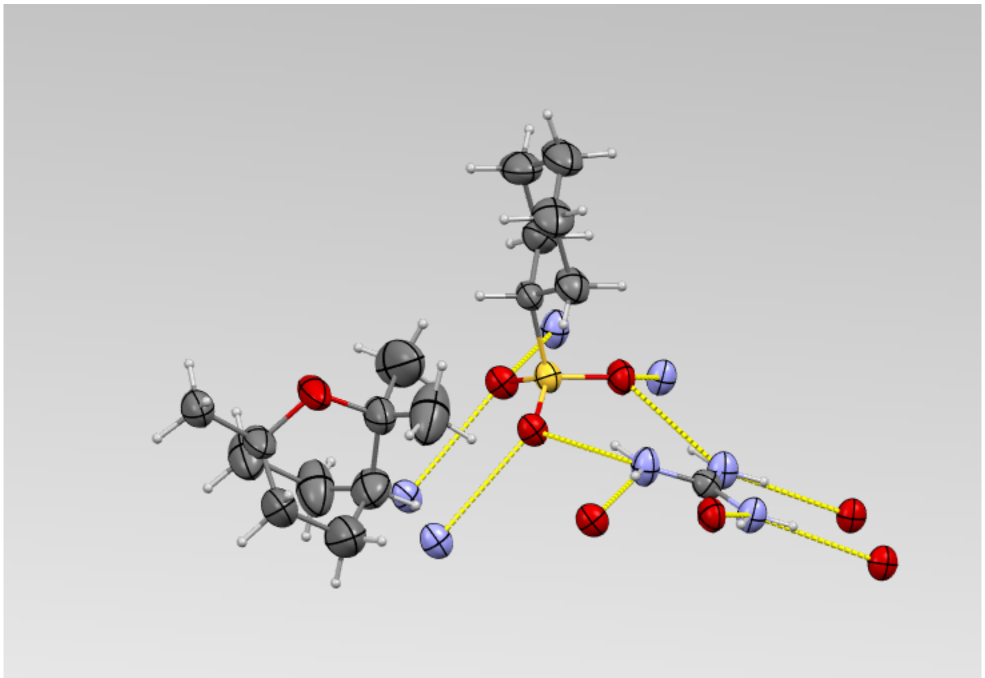

Figure S25. Molecular structure of **25** depicted as ellipsoids with 50% probability. Disordered component removed for clarity.

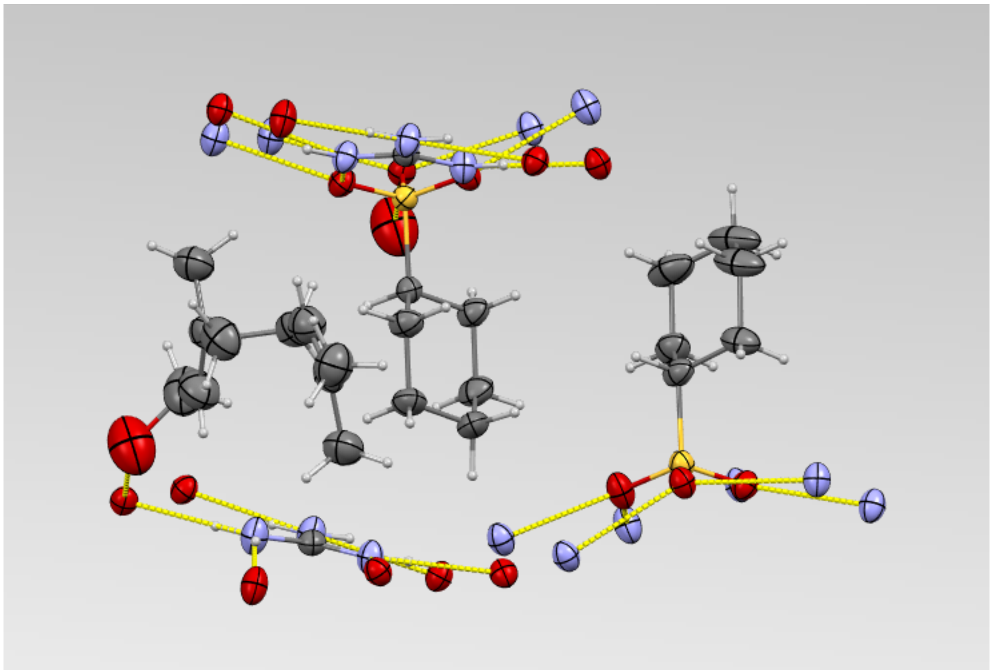

Figure S26. Molecular structure of **26** depicted as ellipsoids with 50% probability.

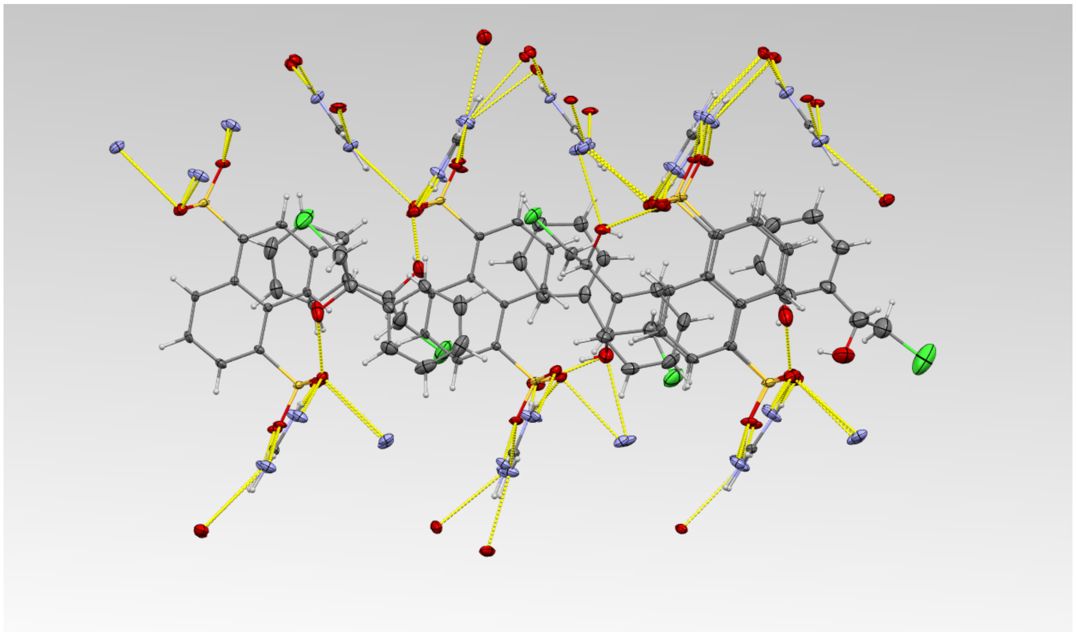

Figure S27. Molecular structure of **27** depicted as ellipsoids with 50% probability. Disordered component removed for clarity.

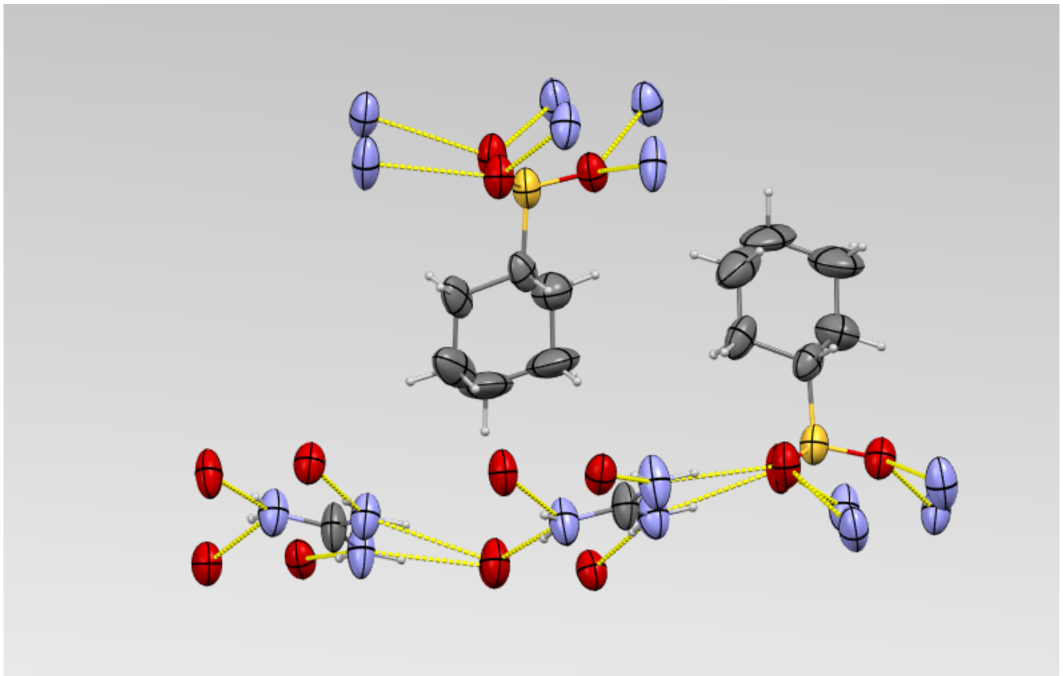

Figure S28a. Molecular structure of **28a** depicted as ellipsoids with 50% probability. Disordered component removed for clarity.

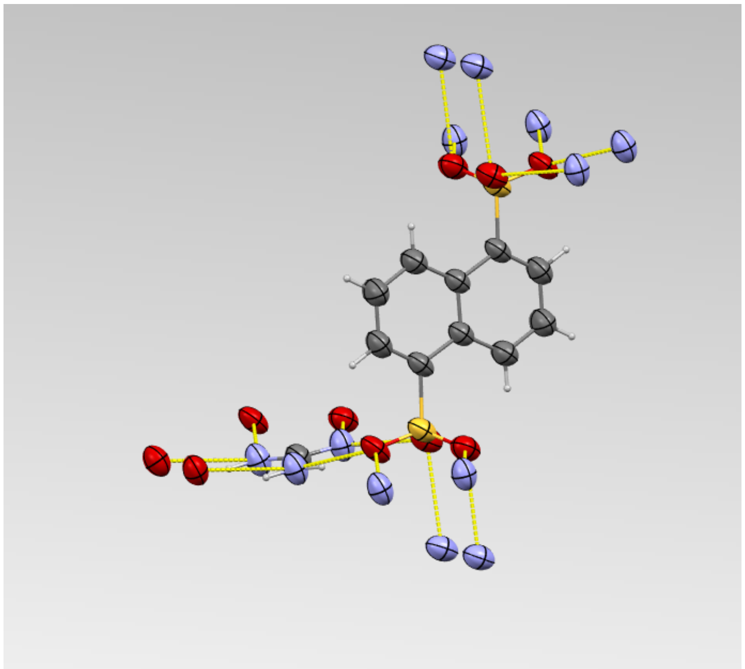

Figure S28b. Molecular structure of **28b** depicted as ellipsoids with 50% probability. Disordered component removed for clarity.

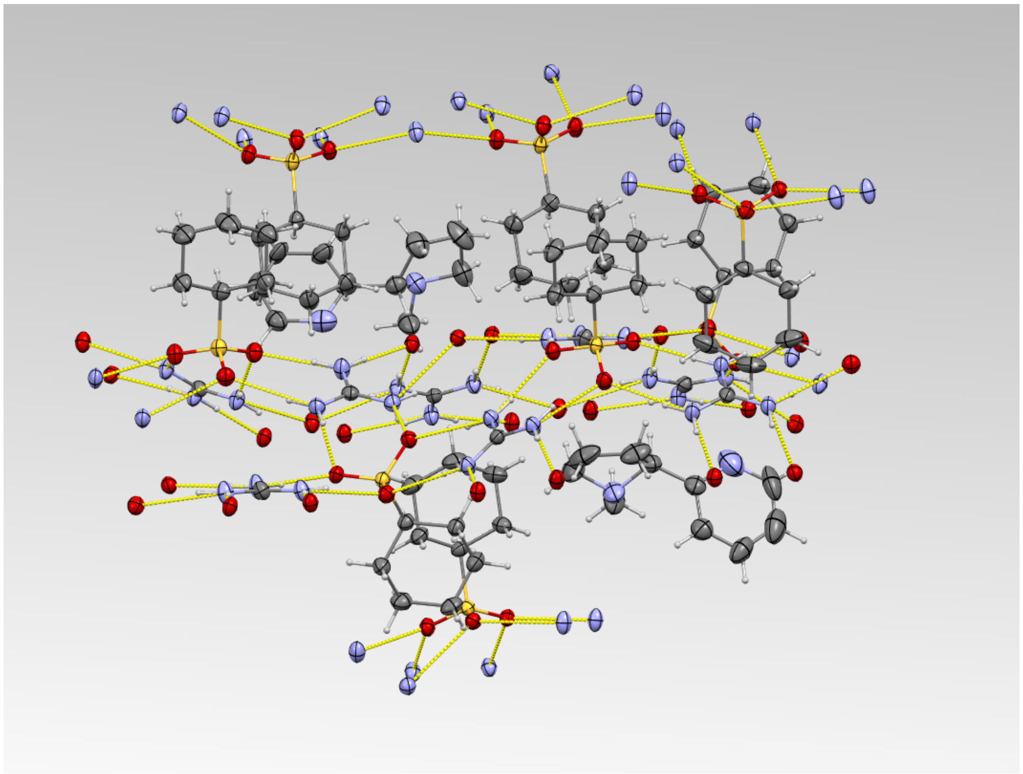

Figure S29. Molecular structure of **29** depicted as ellipsoids with 50% probability. Disordered component removed for clarity.

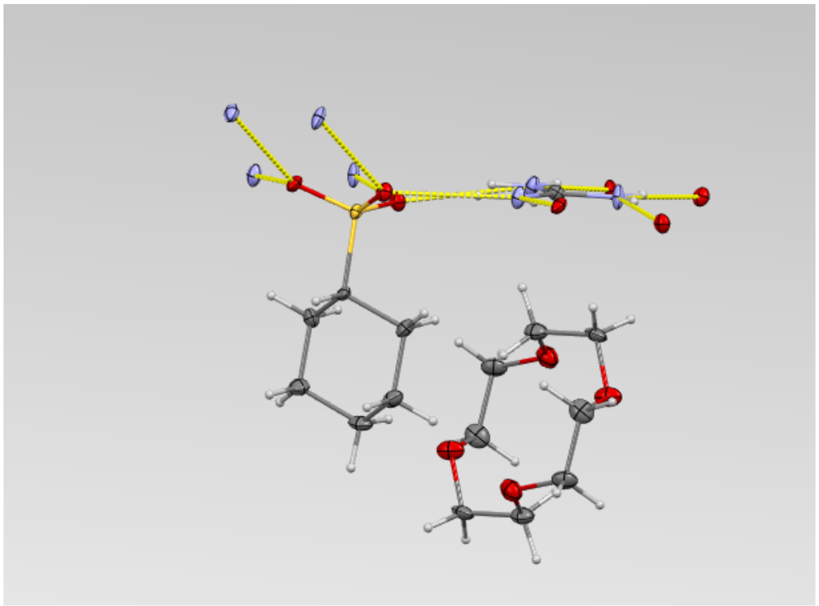

Figure S30. Molecular structure of **30** depicted as ellipsoids with 50% probability. Disordered component removed for clarity.

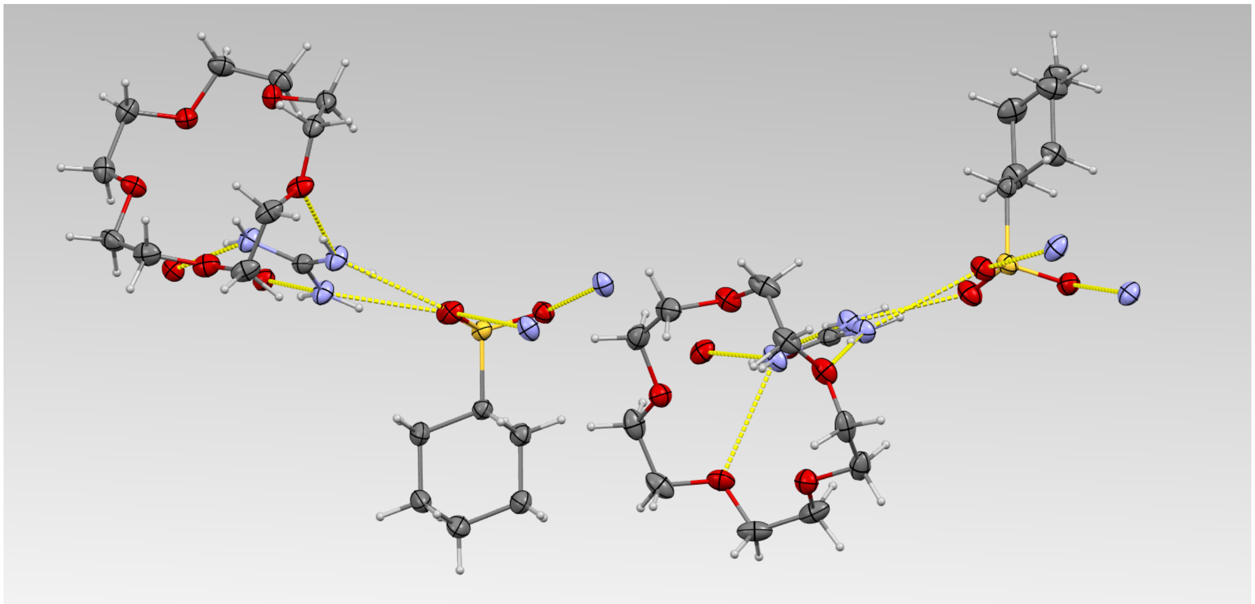

Figure S31. Molecular structure of **31** depicted as ellipsoids with 50% probability.

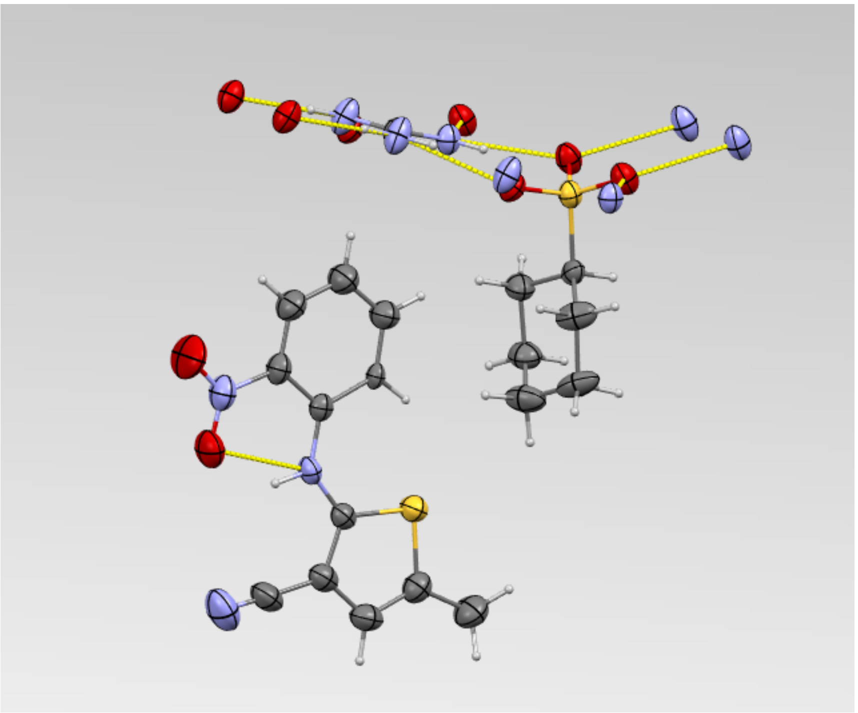

Figure S32. Molecular structure of **32** depicted as ellipsoids with 50% probability. Disordered component removed for clarity.

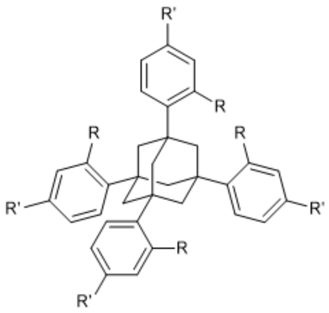

Figure S33. Molecular illustration of the adamantoid chaperones. TDA: R^1 = R' = OMe, TEO: R^1 = R' = OEt, TBro: R^1 = Br, R' = OMe.[13]

Table S3. Summary of the head-to-head comparison of inclusion behavior, R_1 factors and Flack parameters (x) for crystalline inclusion compounds derived from GS hydrogen-bonded hosts and adamantoid molecular chaperones for guest molecules common to both. CCDC deposition numbers for GS inclusion compounds and CSD refcodes for the molecular chaperones are provided for each entry.

		**GS hosts**		**Adamantoid molecular chaperones**		
Inclusion compound	Guest	$G_2$1,5-NDS	GCHMS	TEO	TDA	TBro
**1**	Acetic acid		**2307191**<br>R_1 = 3.13%	"no inclusion"[9]	"no inclusion"[9]	"no inclusion"[9]
**3**	2-butanol		**2307206**<br>R_1 = 6.30%	**TUTXIS**[9]<br>R_1 = 7.07%	**NUJKUA**[12]<br>R_1 = 4.00%	
**4**	(*R*)-2-butanol		**2307219**<br>R_1 = 5.78%<br>x = 0.019(12)	**ZURXES**<br>R_1 = 6.22%<br>x = -0.07(59)		
**5**	Pyridine	**2307201**<br>R_1 = 3.92%		**TUTXUE**[9]<br>R_1 = 5.38%	**ICOZUY**[10]<br>R_1 = 5.29%	"inclusion confirmed" [9]
**7**	(*R*)-2-pentanol	**2307204**<br>R_1 = 4.88%<br>x = 0.046(14)		**ZURXIW**[9]<br>R_1 = 7.35%<br>x = -0.6(6)		
**8S**<br><br>**8**	Aniline	**2307192**<br>R_1 = 4.76%<br><br>**2307202**<br>R_1 = 2.68%		**TUTYIT**[9]<br>R_1 = 5.26%	"no inclusion"[9]	
**9**	(*S*)-2-Hexanol	**2307203**<br>R_1 = 3.93%<br>x = 0.073(13)		**ZURXOC**[9]<br>R_1 = 5.40%<br>x = 0.04(13)		
**16**	1-phenyl-1-propanol	**2307205**<br>R_1 = 5.36%		**RASWER**[11]<br>R_1 = 6.58%		
**17**	(*S*)-(-)-limonene	**2312929**<br>R_1 = 5.07%<br>x = 0.12(6)	Inclusion confirmed, structure could not be resolved due to symmetry issues	**ZURSAJ**[9]<br>R_1 = 4.75%<br>x = 0.2(3)		
**18**	(*R*)-(+)-limonene	Inclusion confirmed, structure could not be resolved due to symmetry issues	**2308623**<br>R_1 = 6.44%<br>x = -0.062(13)	**ZURRUC**[9]<br>R_1 = 4.55%<br>x = 0.07(14)		
**19**	γ-terpinene		**2307214**<br>R_1 = 14.53%	"inclusion confirmed"[9]	"no inclusion"[9]	
**20**	(*R*)-(-)-carvone	**2307189**<br>R_1 = 4.19%<br>x = 0.006(5)		**ZURSEN**[9]<br>R_1 = 4.30%<br>x = -0.04(12)		
**21**	(*S*)-(+)-carvone	**2307190**<br>R_1 = 2.68%<br>x = -0.006(3)		**ZURSIR**[9]<br>R_1 = 4.46%<br>x = 0.21(19)		
**22**	Methyl-L-pyroglutamate	**2307198**<br>R_1 = 2.86%		"no crystals"[9]	"no crystals"[9]	
**23**	(-)-α-thujone		**2307213**<br>R_1 = 13.82%<br>x = -0.05(4)	**ZURSOX**[9]<br>R_1 = 4.91%<br>x = -0.07(20)		

**24S**<br><br>**24**	*cis*-rose oxide		**2307208**<br>R_1 = 4.5%<br>x = 0.12(6)<br><br>**2307217**<br>R_1 = 6.79%<br>x = -0.01(2)	"no inclusion"[9]	"no inclusion"[9]	"no inclusion"[9]
**25**	Eucalyptol		**2307212**<br>R_1 = 11.35%	"inclusion confirmed"[9]	"inclusion confirmed"[9]	
**26**	Geraniol	Inclusion confirmed, structure could not be resolved due to positional disorder	**2307218**<br>R_1 = 4.54%	"no inclusion"[9]	"no inclusion"[9]	**ZURVUG**[9]<br>R_1 = 8.64%
**27**	(*R*)-(-)-2-chloro-1-phenylethanol	**2307195**<br>R_1 = 4.97%<br>x = 0.038(19)		**RASWAN**[11]<br>R_1 = 9.63%<br>x = 0.124(14)		
**28a, 28b**	Eugenol	**2310649**<br>Inclusion confirmed, SQUEEZED data provided	**2307216**<br>Inclusion confirmed, SQUEEZED data provided	**ZURVAM**[9]<br>R_1 = 4.97%		
**29**	Nicotine		**2307209**<br>R_1 = 2.71%<br>x = 0.020(3)	**ZURTAK**[9]<br>R_1 = 4.84%<br>x = 0.00(8)	"inclusion confirmed"[9]	
**30**	12-crown-4		**2307215**<br>R_1 = 6.11%	**ZURTEO**[9]<br>R_1 = 5.43%	**TUTZIU**[9]<br>R_1 = 3.71%	
**31**	15-crown-5		**2307196**<br>R_1 = 5.59%	"no inclusion"[9]		"inclusion confirmed"[9]

Table S4. Comparison of atomic ellipsoids (at 50% probability) for GS host included guests against adamantoid based hosts. If disorder is present, the conformer with greatest occupancy and smallest ellipsoids is shown and disorder is omitted for clarity.

Guest	GS host	Adamantoid host
2-butanol (**3**)	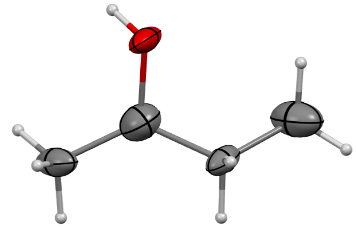	TUTXIS<br>*Could not visualize disorder<br>NUJKUA<br>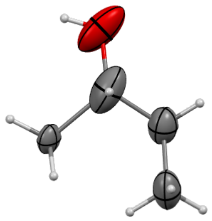
(*R*)-2-butanol (**4**)	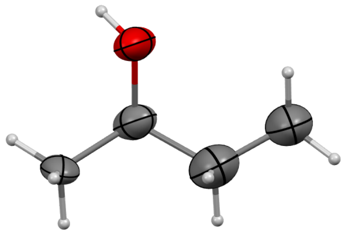	ZURXES<br>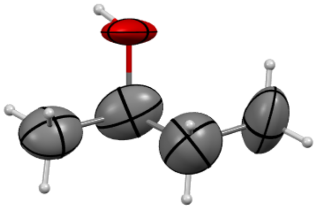
Pyridine (**5**)	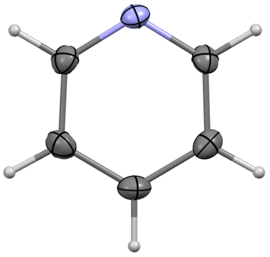	TUTXUE<br>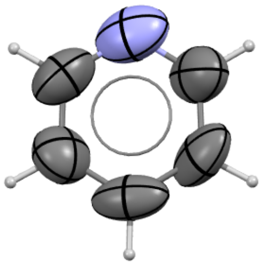<br>ICOZUY<br>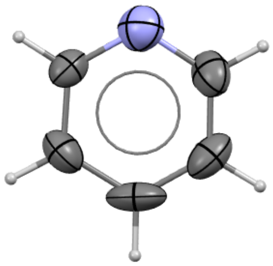

(*R*)-2-pentanol (**7**)	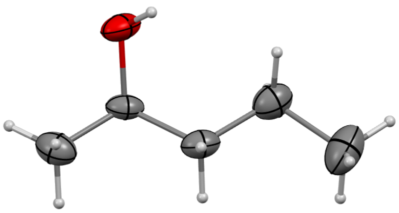	ZURXIW 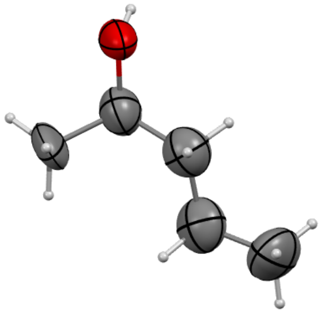
Aniline (**8S, 8**)	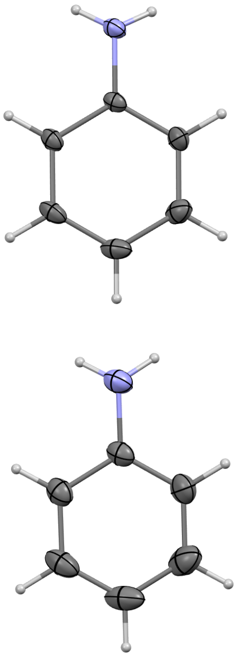	TUTYIT 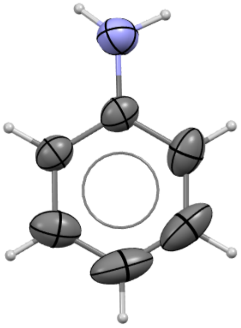
(*S*)-2-hexanol (**9**)	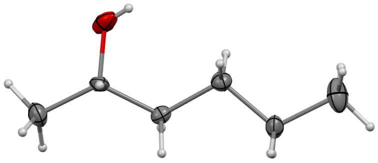	ZURXOC 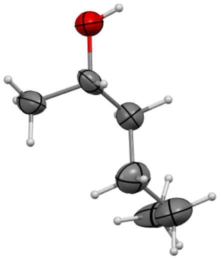

1-phenyl-1-propanol (**16**)	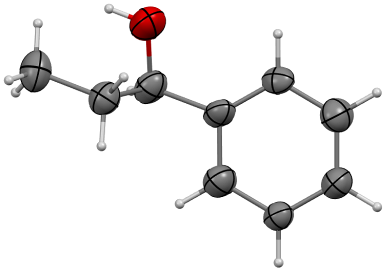	RASWER 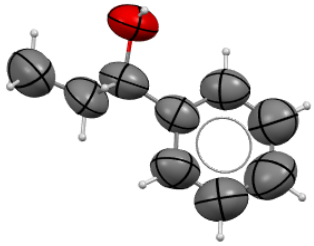
(*S*)-(-)-limonene (**17**)	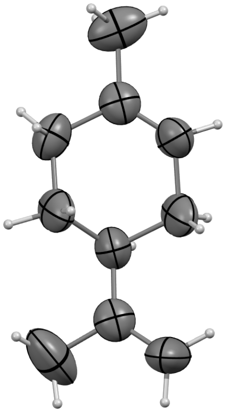	ZURSAJ 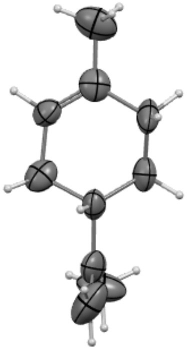
(*R*)-(+)-limonene (**18**)	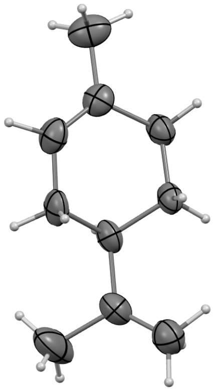	ZURRUC 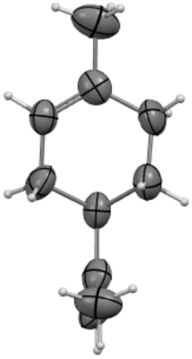

(*R*)-(-)-carvone (**20**)	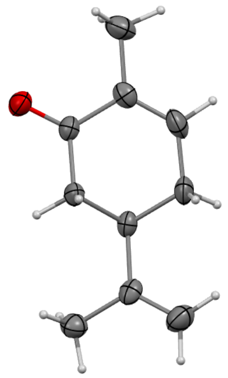	ZURSEN<br>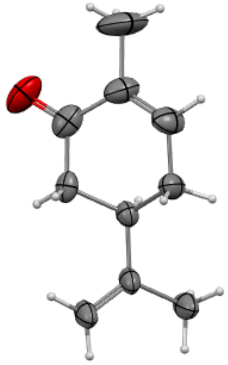
(*S*)-(+)-carvone (**21**)	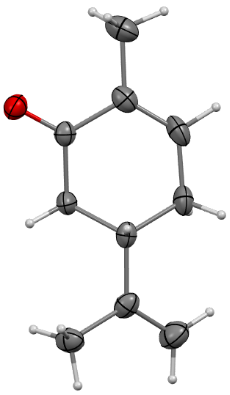	ZURSIR<br>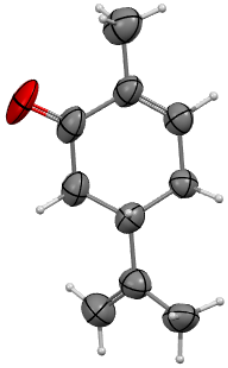
(-)-α-thujone (**23**)	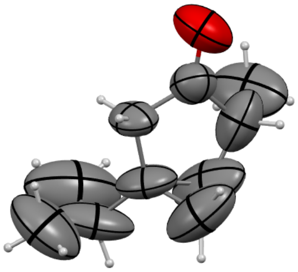	ZURSOX<br>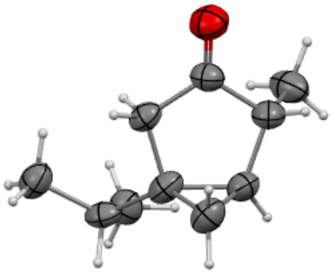
Geraniol (**26**)	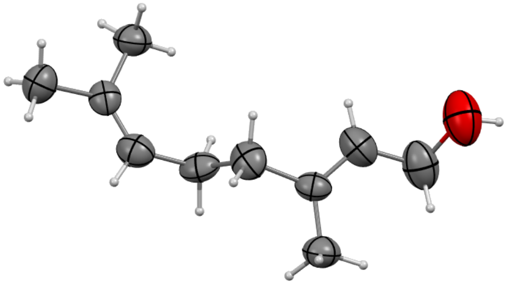	ZURVUG<br>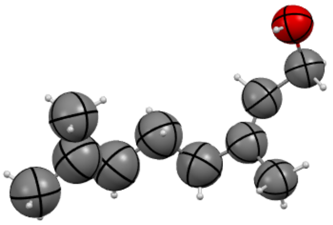

(*R*)-(-)-2-chloro-1-phenylethanol (**27**)	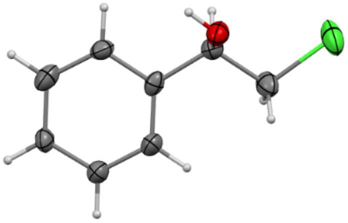	RASWAN<br>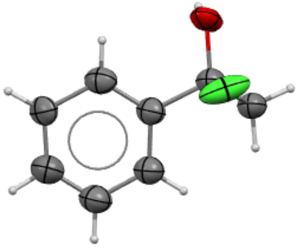
Nicotine (**29**)	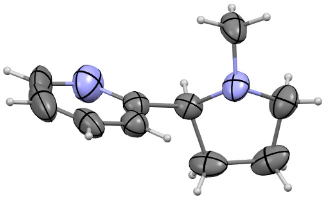	ZURTAK<br>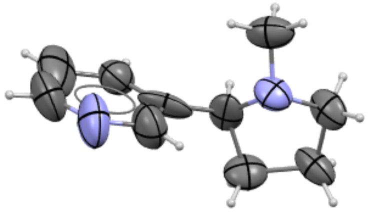
12-crown-4 (**30**)	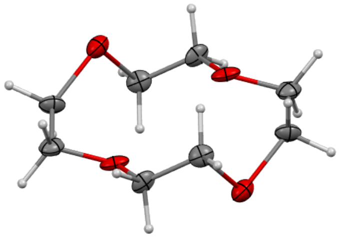	ZURTEO<br>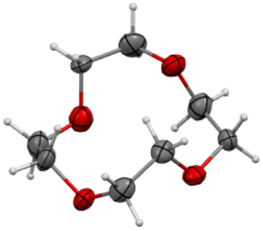<br>TUTZIU<br>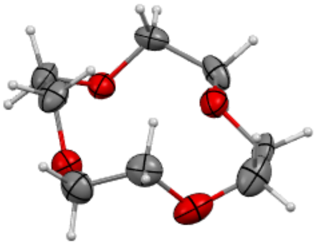

Table S5. Inclusion compounds with $G_2$1,5-NDS, guest volumes, architectures, and puckering angles.

Inclusion compound number	Guest	Guest volume (Å^3)	Architecture	Host:guest ratio	Puckering angle (°)
**1**	acetic acid	58.10	Unique simple brick	1:1	47.70
**2**	tetrahydrofuran	74.19	Unique	1:1	N/A
**5**	pyridine	74.88	Unique simple brick	1:1	64.63
**8**	aniline	92.18	Disrupted bilayer	1:1	N/A
**7**	(*R*)-2-pentanol	103.85	Simple brick	1:1	33.37
**10**	benzonitrile	104.20	Bilayer	2:1	N/A
**13**	1-Cl-3-MeO-propan-2-ol	110.55	Simple brick	1:1	23.92
**9**	2-hexanol	121.15	Simple brick	1:1	38.61
**15**	acetophenone	121.93	Simple brick	1:1	116.03
**12**	R-methylcyclohexanone	123.45	Simple brick	1:1	61.99
**22**	methyl-L-pyroglutamate	132.09	Disrupted simple brick	1:1	46.27
**27**	(*R*)-(-)-2-chloro-1-phenylethanol	139.78	Simple brick	1:1	57.48
**16**	1-phenyl-1-propanol	141.87	Simple brick	1:1	63.13
**17**	(*S*)-(-)-limonene	163.92	Simple brick	1:1	70.10
**28b**	eugenol[a]	165.32	Simple brick	1:1[a]	71.90
**20, 21**	(*R*)-(-)-carvone, (*S*)-(+)-carvone	170.07	Simple brick	1:1	73.14

[a]Host:guest ratio was determined by electron density where guest components could not be modeled fully.

Table S6. Inclusion compounds with GCHMS, guest volumes and host:guest stoichiometries.

Inclusion compound number	Guest	Guest volume (Å^3)	Host:guest ratio
**6**	1,4-dioxane	82.98	2:1
**3, 4**	2-butanol, (*R*)-2-butanol	86.55	2:1
**11**	*cis*-1,2-dimethylcyclohexane	134.60	3:1
**14**	(1-methylcyclohexyl)methanol	143.39	3:1
**25**	eucalyptol	155.91	1:1
**29**	nicotine	160.02	4:1
**23**	(-)-α-thujone[a]	162.99	4:1[a]
**18**	(*R*)-(+)-limonene	163.92	4:1
**19**	γ-terpinene	163.92	2:1
**28a**	eugenol[a]	165.32	2:1[a]
**30**	12-crown-4	169.76	2:1
**24, 24S**	*cis*-rose oxide	175.35	4:1
**26**	geraniol[a]	185.07	2:1[a]
**31**	15-crown-5	213.15	1:1
**32**	ROY	217.61	1:1

[a]Host:guest ratio was determined by electron density where guest components could not be modeled fully.

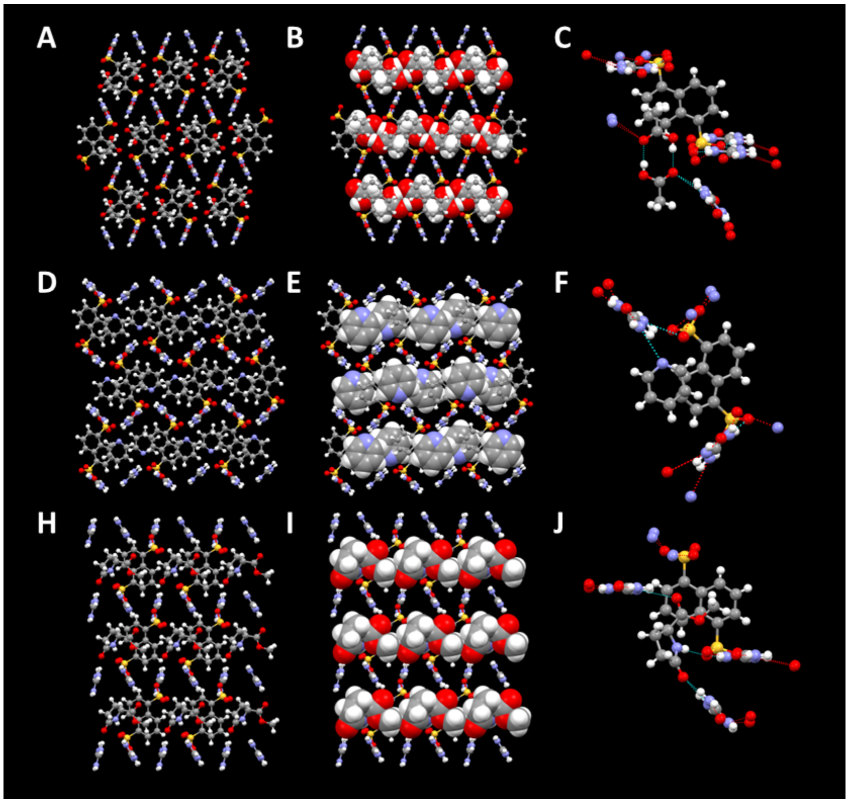

Figure S34. Crystal structures of disrupted simple brick architecture examples (A-C) ($G_2$1,5-NDS)⊃acetic acid (**1**), (D-F) ($G_2$1,5-NDS)⊃pyridine (**5**), and (H-J) ($G_2$1,5-NDS)⊃methyl-L-pyroglutamate (**22**) depicted as ball-and-stick (left panels), with the target guest molecules as space filling (middle panels), and details of the hydrogen bonding between guest and host (right panels).

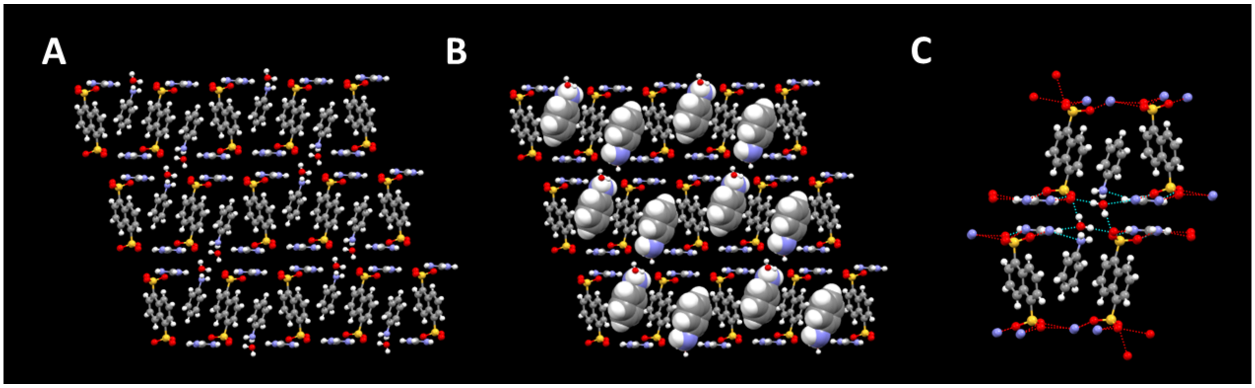

Figure S35. Crystal structure of disrupted bilayer architecture example ($G_2$1,5-NDS)⊃aniline (**8**) depicted as ball-and-stick (A), with the target guest molecules as space filling (B), and details of the hydrogen bonding between guest and host (C).

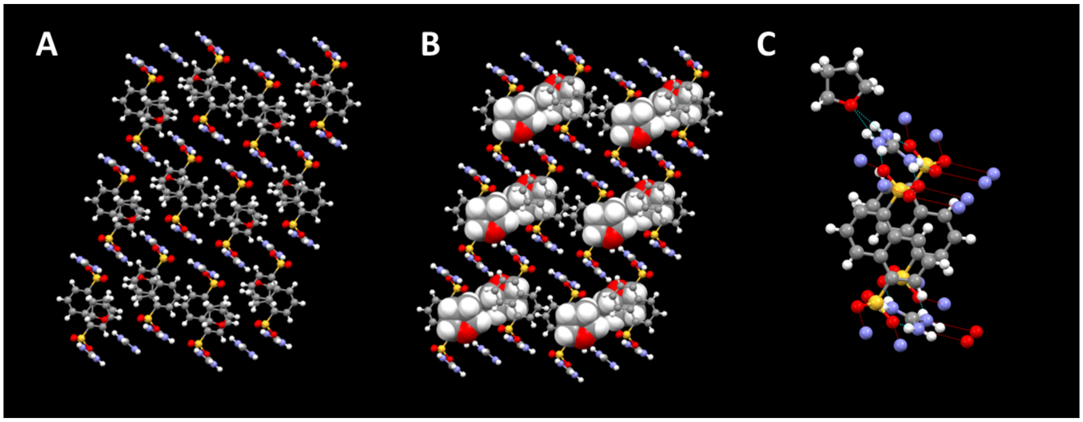

Figure S36. Crystal structure of unique architecture example ($G_2$1,5-NDS)⊃THF (**2**) depicted as ball-and-stick (A), with the target guest molecules as space filling (B), and details of the hydrogen bonding between guest and host (C).

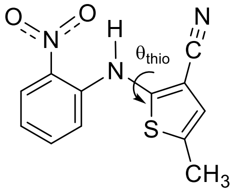

ROY

Figure S37. Molecular illustration of 5-methyl-2-[(2-nitrophenyl)amino]thiophene-3-carbonitrile, also known as ROY, showing the torsion angle of interest, denoted as θ_{thio}.

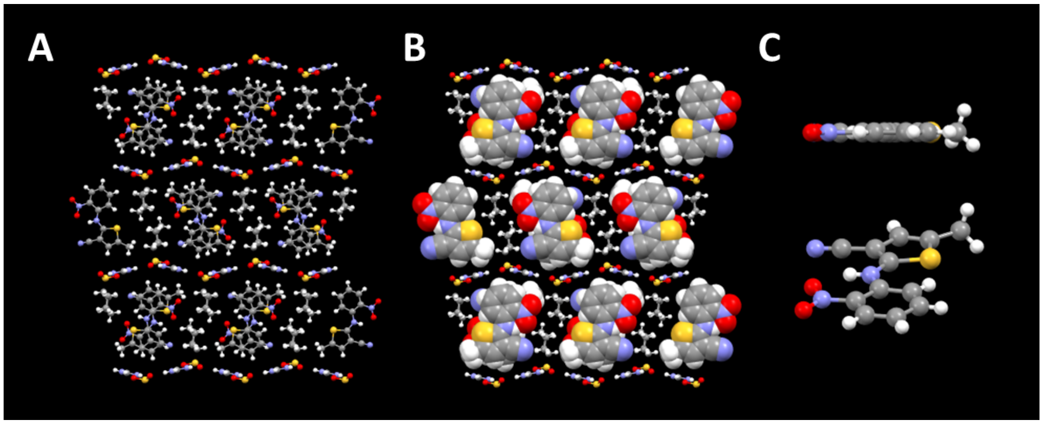

Figure S38. Crystal structure of example (GCHMS)⊃ 5-methyl-2-[(2-nitrophenyl)amino]thiophene-3-carbonitrile, also known as ROY (**32**) depicted as ball-and-stick (A), with the target guest molecules as space filling (B), and the illustration of the planar torsion angle (C).

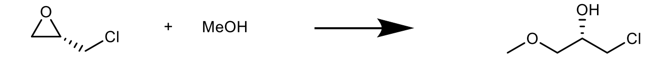

(S)-epichlorohydrin (*S*)-1-chloro-3-methoxypropan-2-ol

Scheme S1. Scheme for the reaction of (*S*)-epichlorohydrin with methanol, affording (*S*)-1-chloro-3-methoxypropan-2-ol.

References

(1) Sigma Aldrich. Cyclohexanesulfonic Acid AldrichCPR 28100-07-4.

(2) Sigma Aldrich. 1,5-Naphthalenedisulfonic Acid 97 211366-30-2.

(3) APEX4. Bruker AXS: Madison, WI 2020.

(4) Sheldrick, G. SHELXT - Integrated Space-Group and Crystal-Structure Determination. *Acta Crystallogr. Sect. A* **2015**, *71* (1), 3–8.

(5) Sheldrick, G. M. Crystal Structure Refinement with SHELXL. *Acta Crystallogr. Sect. C* **2015**, *71* (1), 3–8. https://doi.org/10.1107/S2053229614024218.

(6) Dolomanov, O. V.; Bourhis, L. J.; Gildea, R. J.; Howard, J. A. K.; Puschmann, H. OLEX2: A Complete Structure Solution, Refinement and Analysis Program. *J. Appl. Cryst.* **2009**, *42* (2), 339–341. https://doi.org/10.1107/S0021889808042726.

(7) Macrae, C. F.; Sovago, I.; Cottrell, S. J.; Galek, P. T. A.; McCabe, P.; Pidcock, E.; Platings, M.; Shields, G. P.; Stevens, J. S.; Towler, M.; Wood, P. A. Mercury 4.0: From Visualization to Analysis, Design and Prediction. *J. Appl. Crystallogr.* **2020**, *53* (Pt 1), 226–235. https://doi.org/10.1107/S1600576719014092.

(8) Zhao, Y. H.; Abraham, M. H.; Zissimos, A. M. Fast Calculation of van Der Waals Volume as a Sum of Atomic and Bond Contributions and Its Application to Drug Compounds. *J. Org. Chem.* **2003**, *68* (19), 7368–7373. https://doi.org/10.1021/jo034808o.

(9) Krupp, F.; Frey, W.; Richert, C. Absolute Configuration of Small Molecules by Co-Crystallization. *Angew. Chemie Int. Ed.* **2020**, *59* (37), 15875–15879. https://doi.org/10.1002/anie.202004992.

(10) Schwenger, A.; Frey, W.; Richert, C. Reagents with a Crystalline Coat. *Angew. Chemie Int. Ed.* **2016**, *55* (44), 13706–13709. https://doi.org/10.1002/anie.201605507.

(11) Ou, G.-C.; Chen, H.-Y.; Wang, Q.; Zhou, Q.; Zeng, F. Structure and Absolute Configuration of Liquid Molecules Based on Adamantane Derivative Cocrystallization. *RSC Adv.* **2022**, *12* (11), 6459–6462. https://doi.org/10.1039/D1RA09284G.

(12) Schwenger, A.; Frey, W.; Richert, C. Tetrakis(Dimethoxyphenyl)Adamantane (TDA) and Its Inclusion Complexes in the Crystalline State: A Versatile Carrier for Small Molecules. *Chem. - A Eur. J.* **2015**, *21* (24), 8781–8789. https://doi.org/10.1002/chem.201406568.

(13) Krupp Marie-Idrissa; Frey, Wolfgang; Plietker, Bernd; Richert, Clemens, F. P. Determining the Relative Configuration of Propargyl Cyclopropanes by Co-Crystallization. *Synlett* **2020**, *32* (04), 350–353. https://doi.org/10.1055/a-1293-9867.
